# Muscle differentiation in a colonial ascidian: organisation, gene expression and evolutionary considerations

**DOI:** 10.1186/1471-213X-9-48

**Published:** 2009-09-08

**Authors:** Valentina Degasperi, Fabio Gasparini, Sebastian M Shimeld, Chiara Sinigaglia, Paolo Burighel, Lucia Manni

**Affiliations:** 1 Dipartimento di Biologia, Università degli Studi di Padova, Via Ugo Bassi 58/B, 35131, Padova, Italy; 2 Department of Zoology, University of Oxford, South Parks Road, Oxford OX1 3PS, UK

## Abstract

**Background:**

Ascidians are tunicates, the taxon recently proposed as sister group to the vertebrates. They possess a chordate-like swimming larva, which metamorphoses into a sessile adult. Several ascidian species form colonies of clonal individuals by asexual reproduction. During their life cycle, ascidians present three muscle types: striated in larval tail, striated in the heart, and unstriated in the adult body-wall.

**Results:**

In the colonial ascidian *Botryllus schlosseri*, we investigated organisation, differentiation and gene expression of muscle beginning from early buds to adults and during zooid regression. We characterised transcripts for troponin T (*BsTnT-c*), adult muscle-type (*BsMA2*) and cytoplasmic-type (*BsCA1*) actins, followed by *in situ *hybridisation (ISH) on sections to establish the spatio-temporal expression of *BsTnT-c *and *BsMA2 *during asexual reproduction and in the larva. Moreover, we characterised actin genomic sequences, which by comparison with other metazoans revealed conserved intron patterns.

**Conclusion:**

Integration of data from ISH, phalloidin staining and TEM allowed us to follow the phases of differentiation of the three muscle kinds, which differ in expression pattern of the two transcripts. Moreover, phylogenetic analyses provided evidence for the close relationship between tunicate and vertebrate muscle genes. The characteristics and plasticity of muscles in tunicates are discussed.

## Background

Ascidians (Tunicata) are marine filter-feeding invertebrates that possess a chordate-like swimming larva, which undergoes metamorphosis to form a sessile adult. Typically, they develop three types of muscles during their life cycle: striated in the larval tail, striated in the heart and unstriated in the adult body-wall [1]. The later is commonly called 'smooth muscle', though evidence for homology to vertebrate smooth muscle is weak.

In the larval tail, two bands of mononucleated muscle cells are localised in paraxial position, flanking the notochord and the neural tube. The organisation of their myofibrils resembles that of vertebrate muscle, especially in the arrangement of thin and thick filaments [2-4]. In solitary ascidian embryos, a predetermined number of cells define the muscle lineage (B4.1, A4.1 and b.4.2 blastomeres of the 8-cell stage) [5-8]. Recently, it has been observed that homologues of the transcription factors *MyoD*, *Snail *and *Tbx6*, important regulators of vertebrate myogenesis, are expressed during mesoderm differentiation of ascidian embryos [9,10].

During ascidian embryogenesis, the B7.5 blastomeres give rise to the heart precursors which, after the neurulation, are localised bilaterally and named trunk ventral cells (TVCs) [11]. During metamorphosis, these cells migrate ventrally and contribute to heart tube formation [12]. The myocardium is formed of mononucleated muscle cells with a degree of homology with the heart of vertebrates, in their general structure and the sarcomeric organisation of the myofilaments [13]. At metamorphosis, the larva adheres to a substrate and its tail regresses completely, while the unstriated muscle of the sessile juvenile begins to be recognisable.

The unstriated muscle forms a series of circular and longitudinal bands, which run in the mantle around the oral and atrial siphons and the remaining body-wall. The caudal larval musculature does not contribute to adult body-wall muscle formation. It was proposed that the atrial siphon and circular muscles, together with heart muscle, derive from TVCs, localised laterally to the ventral endoderm [14] and that oral siphon and longitudinal muscles differentiate from trunk lateral cells (TLCs), flanking the visceral ganglion [11]. These muscles have multinucleated cells and do not show any clearly regular sarcomeric arrangement; however, contraction occurs through the troponin/tropomyosin (Tn/Tm) system and is regulated by calcium ions like in striated muscle of vertebrates [15-19]. Moreover, the ascidian body-wall muscle is activated through both muscarinic and nicotinic-type acetylcholine receptors [20-23], as occurs respectively in vertebrate smooth and skeletal muscle [24].

The study of genes coding for proteins associated with the contractile regulatory system can help us to understand the evolution of chordate muscle and the developmental mechanisms in which these genes are involved, and amongst these genes the actins are notable for their highly conserved sequences. Actins are encoded by a multigene family and the expression of each isoform characterises a specific developmental stage or tissue. In several species of solitary ascidians, multiple genes have been characterised that code for both muscle actins (MAs) and non-muscle cytoplasmic actins (CAs) [25-34]. In particular, sequence analyses of cDNA clones have shown at least two different MA isoforms, one in larval muscle and another in body-wall muscle, and while it is not clear which actin is expressed in the heart, it seems to also be different from the larval form [35]. The Tn/Tm regulatory system is common to both vertebrate and invertebrate striated muscle [36], but Tn does not play a role in vertebrate smooth muscle. The troponin complex constitutes TnT, TnI and TnC, which with Tm act as a regulatory switch for striated muscle contraction [37]. Ascidian unstriated muscle has the peculiarity to possess Tns whose presence has been demonstrated both in *Halocynthia roretzi *and *Ciona intestinalis *[15,16,34].

Previous molecular studies of ascidian actins and Tns have all addressed solitary species with studies focused on expression at embryonic stages.

*Botryllus schlosseri *is a colonial ascidian emerging as a model for the study of the developmental mechanisms involved in formation of similar zooids by alternative developmental way, that is the oozooid derived from metamorphosed larva and blastozooid derived from palleal budding [38]. It forms colonies of numerous clonal individuals organised in star-shaped systems embedded in a thin common tunic. Three blastogenetic generations develop synchronously in each colony: the filtering adults, their buds and the budlets of the last generation. Weekly, all the adults regress and are resorbed, while their buds become the new filtering adults and a new generation of budlets is produced. Different to embryonic and larval tissues, which all derive from germ cells, all the blastozooid tissues arise from somatic cells.

In this study, we described the musculature in *B. schlosseri *analysing its organisation, differentiation and gene expression in larvae and in developing blastozooids, beginning from the early bud to the adult and regression stages. We isolated and characterised *B. schlosseri *cDNA clones encoding homologues of a MA (*BsMA2*), a TnT (*BsTnT-c*) and a CA (*BsCA1*); we also obtained the genomic sequences coding for both *BsMA2 *and *BsCA1*, comparing exon-intron organisation to other metazoan actin genes. Phylogenetic analyses with both *BsMA2 *and *BsTnT-c *showed a close relationship between urochordate and vertebrate muscle genes. *In situ *hybridisation (ISH), in parallel with phalloidin staining experiments allowed us to follow the differentiation of the three muscle kinds, which differed in the expression pattern of the two transcripts. The ultrastructure of striated cardiac and unstriated muscle cells was also investigated during the entire blastogenetic cycle from early bud to zooid regression.

## Methods

### Animals and embryos

Colonies of *Botryllus schlosseri *(Styelidae, Stolidobranchia) were collected in the Lagoon of Venice, cultured according to Sabbadin's technique [39] and fed with Liquifry Marine (Liquifry Co., Dorking, England). The transparency of the colonies allowed us to follow the daily development *in vivo *of buds under the stereomicroscope, thereby permitting the selection of appropriate stages. Before utilisation, colonies were staged following Sabbadin's method (see [40]) and anesthetised with MS222 (Sigma) to prevent muscle contractions. Embryos are brooded and mature colonies release swimming tadpole larvae, utilised for the below described *in situ *hybridisation procedure.

### Phalloidin staining

Fragments of colonies were fixed in 4% paraformaldehyde (Sigma) in seawater at 4°C overnight. After washes in phosphate buffered saline (PBS), the fragments were transferred in a buffer solution of PBS containing 1% Triton X-100 (Sigma) at 4°C for 2 h to increase tissue permeability. Subsequently, for F-actin labelling, samples were incubated in a 1:100 phalloidin -FITC or -TRITC conjugated (Sigma) PBS solution, for 2 h in the dark at RT (room temperature). To remove unbound phalloidin conjugate, specimens were washed in PBS and then mounted with Vectashield (Vector Laboratories) and observed. Samples were photographed with a Leica 5000B light microscope accessorised with a Leica DFC 480 digital photo camera and images organised with Adobe Photoshop CS3.

### Transmission electron microscopy (TEM)

Colonies were anesthetised with MS222 and fixed in 1.5% glutaraldehyde buffered with 0.2 M sodium cacodylate, pH 7.4, and 1.6% NaCl. After washing in buffer and postfixation in 1% OsO_4 _in 0.2 M cacodylate buffer, specimens were dehydrated and embedded in Epon 812 resin. Thanks to the transparency of the resin, the specimens were oriented before ultramicrotome cutting. Series of thick sections (1 μm) were stained with toluidine blue and observed to check appropriate levels for preparing ultrathin sections (60 nm), which were given contrast by staining with uranyl acetate and lead citrate. Photomicrographs were taken with a Hitachi H-600 electron microscope (Hitachi High-Technologies Europe GmbH, Krefeld, Germany) operating at 80 V; images were then organised with Adobe Photoshop CS3.

### Identification of transcripts

Partial sequences of cDNAs coding for *BsMA2*, *BsCA1 *and *BsTnT-c *were obtained by screening EST clusters derived from *B. schlosseri *colonies enriched full-length cDNA library (for details see [41]). Resequencing of the *BsMA2 *clones (BMR Genomics) revealed the complete coding sequence, plus part of 5' and 3' UTRs (untranslated region) [EMBL: FN178503]. *BsCA1 *and *BsTnT-c *cDNA clones were incomplete in 5' and 3' regions respectively. The complete transcripts of *BsCA1 *and *BsTnT-c *[EMBL: FN178501 and FN178505] were hence obtained by PCR using, as template, an amplified plasmid library purified pool. The amplification of the library was made following the manufacturer instructions (Creator™ SMART™ cDNA Library Construction Kit, Clontech); the plasmid purification of the pool was obtained using QIAprep Midiprep Kit (Qiagen). The primers were designed (see additional file 1) and used as follows: i) the forward primer pDNR-Lib-FW1 (in the vector sequence) with the reverse primer BsCA-RW1 (in the known partial sequence of the *BsCA1*), ii) one of the forward primers BsTnT-FW1 and BsTnT-FW2 (in the known partial sequence of *BsTnT-c*) with the reverse primer pDNR-Lib-RW1 (in the vector sequence). PCR cycling was performed in a Mastercycler epGradient S (Eppendorf) thermocycler as follow: 94°C for 2', (94°C for 30", 68°C for 30", 72°C for 2') for 35 cycles, 72°C for 8', using Biotaq (Bioline) DNA Polymerase. Ethidium Bromide (Fluka) stained bands from 0.8% agarose gel electrophoresis of the PCRs were subsequently extracted (QIAquick Gel Extraction Kit, Qiagen), cloned into pCRII-TOPO vector (Invitrogen) and sequenced on both strands (BMR Genomics).

### Identification of actin genomic sequences

Genomic DNA from *B. schlosseri *was obtained by incubating a small colony overnight in an extraction buffer (Tris-HCl 50 mM pH 8, EDTA 0.1 mM pH 8, SDS 1%, Proteinase K 0.5 μg/μl). This was followed by: i) centrifugation at 13,000 rpm in a benchtop microcentrifuge, ii) a phenol/chloroform purification and iii) ethanol precipitation and re-suspension in 100 μl H_2_O. PCRs were done using this genomic DNA as template and primers (see additional file 1) designed to the *BsMA2 *and *BsCA1 *sequences obtained: MAgen_R and MAgen_F primers for *BsMA2*, 5'BsCA-gen and 3'BsCA-gen primers for *BsCA1*. Ethidium Bromide stained bands from agarose gel (0.8%) electrophoresis of the PCRs were treated as described previously. After cloning and sequencing, the sequences were identified as the annotated genomic *BsMA2 *and genomic *BsCA1 *[EMBL: FN178504 and FN178502]. All the above mentioned primers were purchased from Sigma-Aldrich.

### Molecular phylogeny and *in silico *prediction

**Figure 1:**
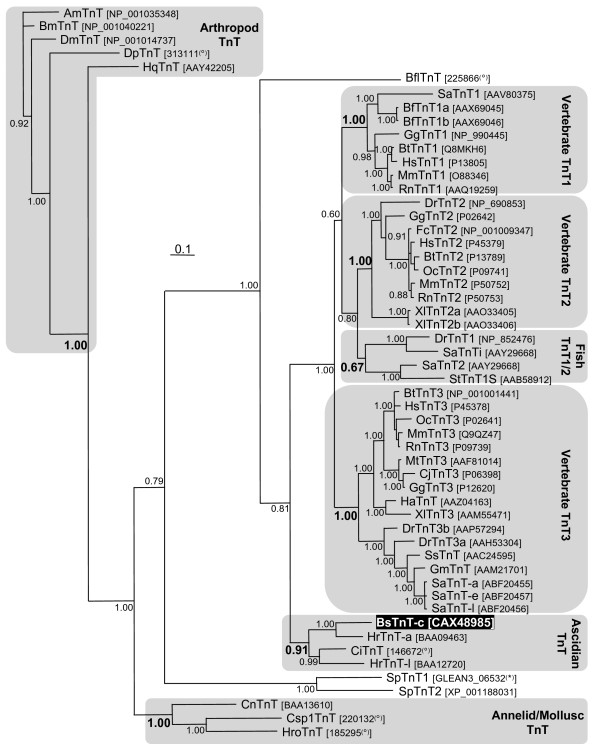
**Molecular phylogenetic analysis of the troponin T protein family**. Bayesian methodology based upon the alignment trimmed (this included removal of both ends, plus some sites in the middle with large insertions in one or a small subset of sequences) as shown in additional file 5. Values adjacent to nodes indicate posterior probabilities. Some inferred groupings are boxed in grey, labelled to the right of the figure and with supporting value in bold. The vertebrate sequences fall into three groups reflecting the well-characterised TnT1, TnT2 and TnT3 genes, with a fourth group (labelled Fish TnT1/2) containing only bony fish sequences, the relationship of which to other TnT groups cannot be determined. The *B. schlosseri *(BsTnT-c, black highlighted) sequence falls into a group of ascidian sequences. Note that another ascidian, *H. roretzi *(Hr), has two TnT protein, and the *B. schlosseri *sequence is most closely related to the adult (HrTnT-a) one. Protostome sequences have been used to root the tree, as TnT genes have not been identified outside the bilateria. The positioning of echinoderm (*S. purpuratus*; Sp) and amphioxus (*B. floridae*; Bfl) sequences reflects the likely phylogenetic relationships of these taxa. Accession numbers of sequences are shown in brackets adjacent to the protein name (^(*) ^and ^(°) ^are downloaded from HGSC and JGI database respectively, all the others are from GenBank). Other abbreviations: -a, adult; -e, embryonic; -l, larval; Am, *Apis mellifera*; Bf, *Bufo marinus*; Bm, *Bombyx mori*; Bt, *Bos taurus*; Ci, *Ciona intestinalis*; Cj, *Coturnix japonica*; Cn, *Chlamys nipponensis*; CspI, *Capitella *sp. I; Dm, *D. melanogaster*; Dp, *Daphnia pulex*; Dr, *Danio rerio*; Fc, *Felis catus*; Gg, *Gallus gallus*; Gm, *Gadus morhua*; Hc, *Hyla chrysoscelis*; Hq, *Haemaphysalis qinghaiensis*; Hro, *Helobdella robusta*; Mm, *Mus musculus*; Mt, *Mitu tomentosa*; Oc, *Oryctolagus cuniculus*; Rn, *Rattus norvegicus*; Sa, *Sparus aurata*; Ss, *Salmo salar*; St, *Salmo trutta*; Xl, *Xenopus laevis*.

Alignments and pairwise identity scores were constructed with CLUSTALW2 on the EMBL-EBI site [42-44]. using default parameters on datasets comprising the *B. schlosseri *predicted amino acid sequences deposited in EMBL. Sequences from other species were downloaded from GenBank, JGI or HGSC (see additional files 2, 3 and Figure 1 for accession numbers). Bayesian phylogenetic analysis was conducted in MrBayes3.1 (default settings [45,46]) using the aligned complete amino acidic sequences of 65 actins and the trimmed alignment of 54 complete amino acidic sequences of TnTs (see supplementary material for alignments). The analysis was continued for 1 million generations, examined for convergence and the first 25% discarded when compiling summary statistics and consensus trees. Phylogenetic trees were viewed in Treeview [47] and then imported into PowerPoint for labelling. NetPhosK and NetAcet [48,49] were used to improve prediction of kinase specific protein phosphorylation sites and substrates of N-acetyltransferase A respectively. Determination of exon-intron arrangements of *BsCA1 *and *BsMA2 *genes was performed by aligning mRNA to genomic sequences with Spidey tool [50].

### *In situ *hybridisation (ISH)

An RNA probe for *BsMA2 *was obtained by cloning the original insert of the cDNA library clone in pGEM-3Z vector (Promega); the probe, coding for a 1375 bp specific region, comprises the complete annotated transcript. RNA antisense probe for *BsTnT-c *was obtained from a clone in pCRII-TOPO vector, resulting from the PCR described above on the amplified cDNA pool; the probe comprises a 1004 bp specific 3' region, and includes the entire coding sequence of the annotated transcript. According to the protocol supplied with the DIG RNA Labelling kit (Roche Molecular Biochemicals) and the vectors, an appropriate restriction enzyme (*Not*I or *Hin*dIII, Promega) and T7 or SP6 RNA polymerase (Promega) were used to linearise the vector and obtain antisense probe.

Specimens for ISH (*B. schlosseri *colonies or mature larvae) were fixed overnight in freshly prepared MOPS buffered (0.1 M MOPS (Sigma), 1 mM MgSO_4 _(Sigma), 2 mM EGTA (Fluka), 0.5 M NaCl) 4% paraformaldehyde (Taab). Fixative was removed by washing twice in PBS pH 7.4 (Oxoid) and then samples were dehydrated through graded PBS/Ethanol to 100% then washed in xylene and embedded in Paraplast Plus (Sherwood Medical). Samples were serially sectioned (12 μm) and left to adhere to microscope slides, cleaned from the Paraplast with xylene (15 min), rehydrated in a graded series of ethanol to PBS, then used immediately. Sections were incubated (6 min) in 10 μg/ml proteinase K (Promega) in PBS; the enzyme action was then stopped with a solution of 0.2% glycine in PBS, washed in PBS, postfixed in a 4% paraformaldehyde plus 0.2% glutaraldehyde solution in PBS and re-washed in PBS. Samples were then incubated in the hybridisation mix (50% formamide (Fluka), 1% Blocking Reagent (Roche), 5 mM EDTA (Fluka), 0.1% Tween-20 (Sigma), 0.1% CHAPS (Roche), 1 mg/ml heparin (Sigma) and 1 mg/ml tRNA (Roche), SSC 5×) 1 h at 65°C and after overnight with 1-2 μg/ml DIG-labelled riboprobes. Specimens were washed twice in 2× SSC pH 4.5, three times in formamide 50% in 2× SSC pH 4.5 30 min at 65°C, and twice in PBS-T (0.1% Tween-20 in PBS). Subsequently slides were: i) incubated in a blocking solution (2% Blocking Reagent, 10% goat serum (Sigma) in PBS-T) 1 h at RT and then overnight with an alkaline phosphatase conjugate anti-DIG-antibody (Roche) for riboprobe detection, ii) treated with a NBT/BCIP solution (Roche) as alkaline phosphatase substrate, until the dye was detectable, iii) dehydrated in ethanol to a final step of xylene (15 min) and mounted in Eukitt (Electronic Microscopy Sciences). Sections were photographed with a Leica 5000B light microscope accessorised with a Leica DFC 480 digital photo camera; images were then organised with Adobe Photoshop CS3.

## Results

### Isolation and characterisation of *BsMA2, BsCA1 *and *BsTnT-c*

From a full-length cDNA library derived from *B. schlosseri *colonies we isolated three clones that a BLAST search identified as encoding probable muscle-type actin, cytoplasmic-type actin and troponin T respectively. The three cDNA clones each include the complete predicted open reading frame (ORF), plus part of the 5' and 3' untranslated regions (UTRs). Moreover, genomic sequences encompassing the exons encoding the complete ORF of both actin genes were isolated from genomic DNA obtained from *B. schlosseri *colonies.

#### Actins

The two actin cDNA clones are 1375 and 1700 bp long, with ORFs of 1140 and 1131 bp from which we deduce respective sequences of 379 and 376 amino acids. The predicted amino acid sequences are aligned with actins of other metazoans and specific amino acid residues distinctive for vertebrate muscle and cytoplasmic actin forms are compared [51,52] (see additional file 4 for a schematic view; see additional file 5 for the complete alignment). As shown in additional file 4, the sequence that shares 13 of 20 diagnostic residues in common with the mammalian α-skeletal actin is named *BsMA2 *(***B***. ***s****chlosseri ***M**uscle **A**ctin **2**) and is coding for a muscular actin form. The other sequence is more related to the vertebrate cytoplasmic actins, so we named it *BsCA1 *(***B***. ***s****chlosseri ***C**ytoplasmic **A**ctin **1**). We note that a region of the transcript here characterised was identified in a previous work [53].

The alignment analysis reveals that *BsMA2 *is characterised by the same amino acid residues as the ascidian adult muscle actins at diagnostic positions (103, 176 and 272). The amino-terminal region is highly variable between *BsMA2 *and *BsCA1*, as was found in other species (additional file 6). The adult muscle actin of *B. schlosseri *(*BsMA2*) lacks the Cys residue after the first Met and possesses a series of six acidic amino acids (D and E). This is consistent with the situation of the other chordates, where the muscle actins are characterised by at least four acidic residues in the first positions.

The amino-terminal of *BsCA1 *has a Cys next to the first Met like the non-chordate cytoplasmic actins and lacks both the typical acidic amino acids and additional residues that characterise the muscle isoforms (Thr^6^, Cys^10^, Leu^16 ^and Val^17^; additional file 4).

#### Troponin T

The third cDNA clone isolated from the cDNA library is 1103 bp long and encodes for a predicted protein of 261 amino acids, deduced from an open reading frame of 786 nucleotides. An alignment with other isoforms of troponin T isoforms showed the presence of specific amino acids in conserved positions (see additional file 7). We hence named the clone *BsTnT-c *(***B***. ***s****chlosseri ***T**roponin **T-c**). The TnTs of both vertebrates and ascidians are normally composed of a single polypeptide chain of about 250 to 300 residues, whereas the invertebrate forms exhibit an additional polar C-terminal extension of about 100 amino acids [54]. The N-terminal displays a series of acidic residues, rich in glutamic acid. BsTnT-c also has this glutamic acid rich N-terminal region, though it is shorter than that in most vertebrates. Ser^2^, the first residue after methionine, is normally acetylated and phosphorylated by casein kinase II (CKII) in the rabbit skeletal muscle TnT isoform (OcTnT_2f_) [55,56]. BsTnT-cpossesses both the Ser^2 ^and a sequence that is compatible with the consensus site of casein kinase II (**S**^2^*XX***E**, additional file 7) [57]. This also conforms to NetPhosK and NetAcet software predictions (see methods) of BsTnT-c Ser^2 ^residue as phosphorylated and acetylated site with a score of 0.71 and 0.52 respectively. The alignment with other ascidian and vertebrate TnTs reveals that the central region of the protein is more conserved than the N- and C-terminals and probably contains the interaction site for Tm (see additional file 7) [58-60]. Taking into consideration what it is known in vertebrates, we found that regions displaying a certain degree of similarity with other components of the troponin complex, such as Tm, TnC and TnI (see additional file 8) are recognisable by comparison and demonstrated to be more conserved [[54,58,59,61] and [62]].

The analysis extended to the entire length of the sequence shows that the two ascidian species possess a TnT that is more similar to OcTnT_2f_, a vertebrate isoform, with respect to the cephalochordate and echinoderm TnTs. This pattern is also maintained when we consider only the regions subject to the interaction with other proteins.

### Exon-intron organisation of *BsMA2 *and *BsCA1*

Fragments of genomic DNA corresponding to both *BsMA2 *and *BsCA1 *were amplified by polymerase chain reaction (PCR) and subsequently sequenced. We obtained clones of 1975 and 1752 nucleotides, corresponding respectively to the entire coding region for the *BsMA2 *and to the coding region together with part of the 3' UTR for *BsCA1*. The exon-intron organisation, analysed between the start and stop codons, revealed the presence of four introns in *BsMA2*, which interrupt the sequence at the amino acid positions 45-3, 153-1, 208-2 and 271-1 (additional file 2; numbers after the amino acid residue indicate codon phase number of intron). Six intron positions are present in *BsCA1 *(42-3, 114-3, 150-3, 204-1, 247-3 and 308-1; additional file 2), as previously reported in the ascidian *HrCA1 *[35].

### Phylogenetic analysis of actin and troponin T sequences

The deduced amino acid sequences of *BsCA1*, *BsMA2 *and *BsTnT-c *were used for the molecular phylogenetic analyses. The Bayesan phylogenetic tree for actins (see additional file 3) results in many nodes poorly supported or collapsed. The cytoplasmic actins together with the non-chordate invertebrate forms are separated from the chordate muscle isoforms. BsCA1 and BsMA2 fall to the first and the second groups respectively. Further, BsMA2 is placed in a robustly supported group of ascidian adult MAs. Interestingly, ascidian larval MAs form a robustly supported separate group.

A Bayesian phylogenetic tree was also constructed for TnT (Figure 1). In this tree BsTnT-c groups with the other ascidian TnTs, and in particular is most related to the adult TnT of *H. roretzi*. Ascidian TnTs group with vertebrate TnTs, when the tree is rooted with protostome TnTs. Vertebrate TnTs reflect the well-characterised skeletal-slow (TnT1), cardiac (TnT2) and skeletal-fast (TnT3) groups, with a fourth poorly supported group (posterior probability 0.67) containing only bony fish sequences.

### General organisation of musculature in the adult blastozooid

#### Body-wall

**Figure 2:**
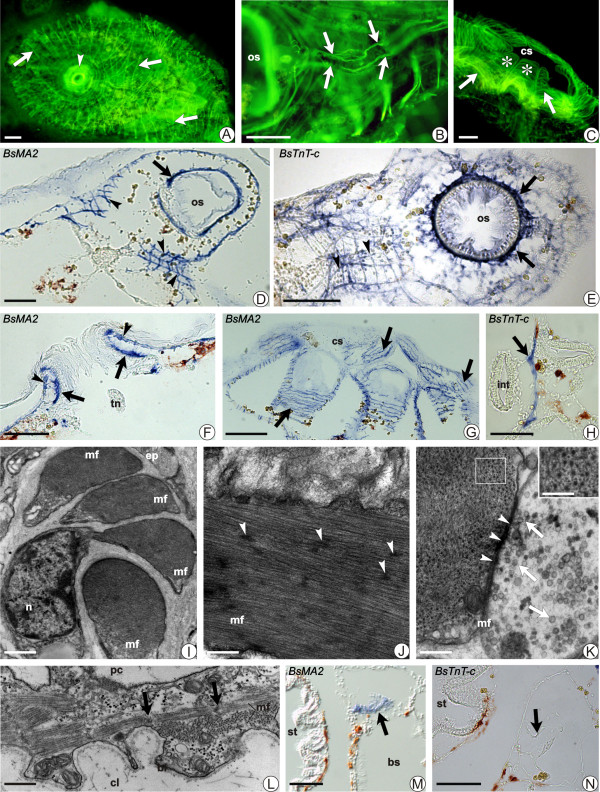
**Aspects of the adult blastozooid musculature of *B. schlosseri***. Phalloidin -FITC staining (A-C), ISH onsection (D-H; M, N) and TEM (I-L). **A) **Dorsal view; general organisation of musculature (arrows). Arrowhead: oral siphon. Scale bar = 0.1 mm. **B) **Two longitudinal fibres (arrows) accompany the dorsal lamina. (os), oral siphon. Scale bar = 50 μm. **C) **Cloacal siphon (cs)formed by the confluence of dorsal languets (*). Arrows: musclefibres. Scale bar = 0.1 mm. **D, E) **Tangential sections. Muscle fibres (arrows) around the oral siphon (os). ISH of *BsMA2 *(D) and *BsTnT-c *(E). Arrowheads: transverse fibres. Scale bar = 50 μm. **F) **Oral siphon: inner (arrows) and outer (arrowheads) systems of fibres (*BsMA2*). (tn) tentacle not stained. Scale bar = 50 μm. **G) **Tangential section of cloacal siphon (cs) of a system (*BsMA2*). Arrows: dorsal languets with muscle fibres (arrows). Scale bar = 0.15 mm. **H) **Fibre (arrow) in therecto-oesophageal trabecula (*BsTnT-c*). (int), intestine. Scale bar = 50 μm. **I) **Transverse section of unstriated muscle fibres (TEM). Muscle cellsare polarised. (mf), contractile elements; (ep), epidermis; (n), nucleus. Scale bar = 0.75 μm. **J) **Longitudinal section of an unstriated muscle fibre (mf). Arrowheads: dense bodies. Scale bar = 0.2 μm. **K) **Neuromuscular junction. Small vesicles (arrows) fill the nerve termination; arrowheads: dense material in the junctional space. In transverse section of an unstriated fibre (mf), no regular disposition of filaments is recognisable (inset; scale bar = 0.09 μm). Scale bar = 0.18 μm. **L) **Longitudinal section of myocardium. Sarcomeres are defined by arrows. Thin basal lamina (bl) faces the cardiac lumen (cl). (mf), myofibrils; (pc), pericardial cavity. Scale bar = 1 μm. **M, N) **Cardiac area (arrow). ISH of *BsMA2 *labels myocardium (M); no signal for *BsTnT-c *expression (N). (bs), branchial sac; (st), stomach. Scale bar = 50 μm.

**Figure 3:**
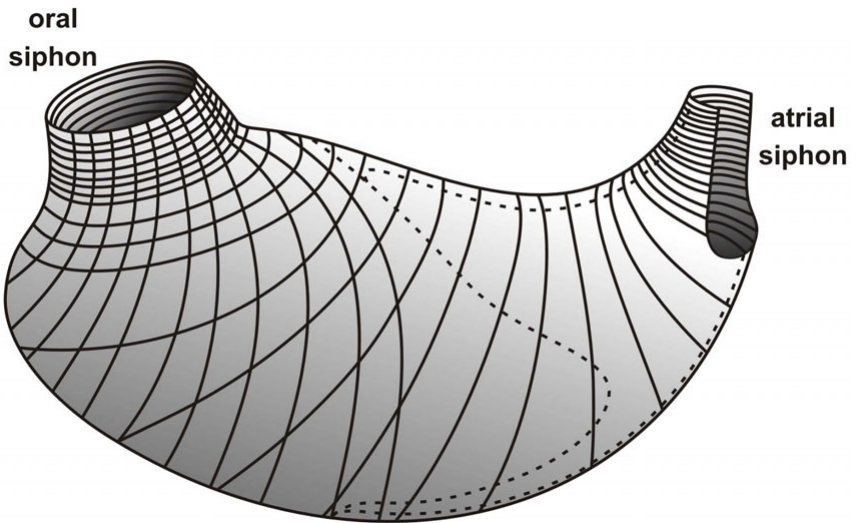
**Representation of the musculature of a blastozooid of *B. schlosseri***. On the left is shown the oral siphon, while at the opposite site is atrial siphon. Filaments of body-wall musculature are shown, starting from the siphons.

We describe the musculature in the colonial ascidian *B. schlosseri*, analysing muscle organisation in the adult blastozooid (Figure 2A-N) using phalloidin-staining, ISH on sections and TEM. In blastozooids stained with phalloidin-FITC, superficial muscles are organised in a tightly packed network of circular and longitudinal fibres, which run throughout the whole body-wall (Figures 2A-C). Circular fibres are mainly concentrated around the siphons, whereas the longitudinal ones constitute muscles that depart from the external wall of the oral siphon to the lateral region of the zooid. The combination of phalloidin staining and ISH against the *BsMA2 *and *BsTnT-c *mRNAs (Figures 2D-H) permitted a detailed reconstruction of the arrangement of muscle fibres (Figure 3). An inner and an outer fibre system are evident at the oral siphon level. The outer system is formed by a series of about twenty bundles that surround the opening, with a concentric disposition from the apical portion of the siphon to the base of velum. From this point about ten fibres extend in the direction of the pericoronal bands, until the conjunction between the branchial sac and mantle: radial bundles originate from the oral siphon reaching a perpendicular disposition with the circular fibres, both forming a regular network of filaments in the anterior and lateral region of the zooid (Figures 2A, D-E).

A complex of numerous transverse bundles forms the inner muscular system, placed around the siphon opening. At the base of the siphon, muscle fibres concentrate around the velum but do not penetrate into the tentacles (Figure 2F) [63]. The common cloacal siphon forms after the confluence of the atrial siphons of each zooid, and possesses a dense musculature (Figures 2C, G). At this level, the transverse fibres appear more packed and surround the atrial siphon, extending subsequently along the dorsal languets, which are connected to each other to constitute the common cloacal chamber (Figures 2C, G). The internal organs do not show signs of musculature, except for two longitudinal fibres that depart from the neural complex area, move down along the dorsal lamina and run in the mantle through the gastric trabecula (Figures 2B, H). Anteriorly, fibres elongate around the oral siphon, following the pericoronal sinuses border (Figure 2B).

The ultrastructural organisation of musculature shows that all the unstriated muscle fibres share similar features in each part of the zooid. The cytoplasm is filled with contractile material (Figure 2I) and most cell organelles, such as nuclei and mitochondria, are confined in peripheral regions of the fibres. The contractile material consists of thin and thick filaments closely apposed, and in longitudinal sections do not reveal any sarcomeric organisation (Figure 2J). However, occasionally in transverse sections the thick filaments appear surrounded by thin filaments with the arrangement recalling that of the striated muscle (Figure 2K, inset). Dense bodies are recognisable in longitudinal sections along the fibre, representing regions to which thin and thick filaments bind. The disposition of these dense bodies is not random, however it was not possible to establish if they are distributed according a specific pattern (Figure 2J).

Fibres are covered by fuzzy material forming a thin basal lamina. In some cases, contiguous fibres present their sarcolemma closely apposed to each other and the extra-cellular material is no longer recognisable. Nervous fibres contact single muscle cells or penetrate between apposed fibres and form neuromuscular junctions (Figure 2K), which have been previously described as acetylcholinesterase (AChE) positive [64].

#### Heart

The heart of *B. schlosseri *is located in the ventral mantle and extends between the posterior limit of the endostyle and the stomach. It is in form of a curved double walled tube of external pericardium and internal myocardium, connected to each other at the level of the longitudinal rafe. The myocardium is responsible for the reversible, helicoidal contraction that drives blood movement. Phalloidin staining allows us to recognise muscle fibres at the myocardium level, organised in a dense network of transverse thin bundles. At the ultrastructural level (Figure 2L) the myocardic cells appear polarised, with the contractile material organised in striated myofibrils close to the cardiac lumen. The sarcomeric distribution and the ratio 1:2 of thick to thin filaments recalls the striated musculature of the larva [65]. In many sections it is possible to see a deeper layer of myofibrils oriented in a right angle with respect the superficial one. ISH for the localisation of *BsMA2 *transcripts show signal at the myocardial level, localised between stomach and branchial sac (Figure 2M). No *BsTnT-c *expression was found in the heart of the adult zooid (Figure 2N).

### Musculature differentiation during the blastogenetic cycle

#### Body-wall

**Figure 4:**
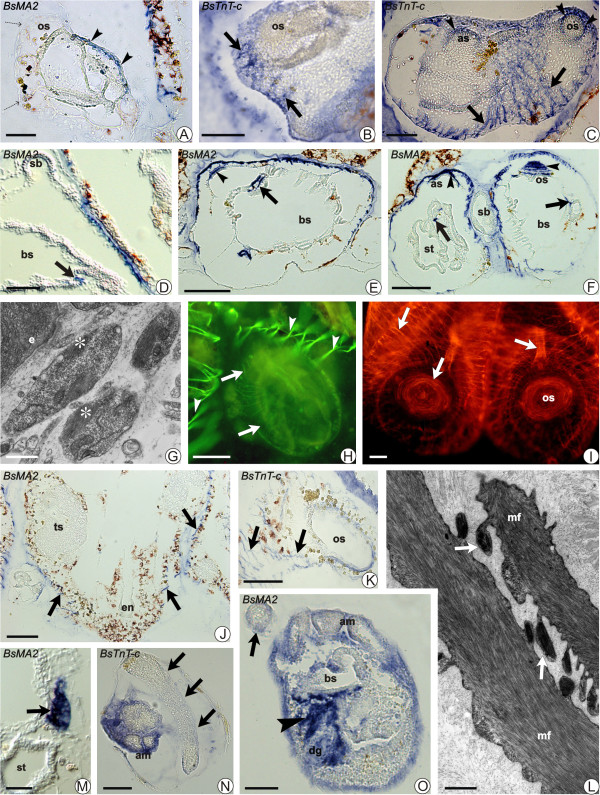
**Modifications of the musculature during the blastogenetic cycle**. Bud development (A-I; M), take-over (J-L), and larval phases (N, O), shown by: ISH on sections (A-F; J; K; M-O), phalloidin -FITC (H), -TRITC (I), and TEM (L). **A) **Stage 6; signal in the intersiphonal region (*BsMA2*; arrowheads). (os), oral siphon. Dotted arrows: epidermis. Scale bar = 50 μm. **B) **Stage 7; myoblasts (arrows) around the rudiment oforal siphon (os). Scale bar = 50 μm. **C-F) **Stage 8; mantle fibres between oral (os) and atrial (as) siphons (C, ISH for *BsTnT-c*). Fibres run along the dorsal lamina (arrows, E, F) and through the recto-oesophageal trabecula (arrows, D, F). Arrowheads (C, E, F): fibres around the siphons. (bs), branchial sac;(st), stomach; (sb), secondary bud. Scale bar = 50 μm (C, D); scale bar = 0.1 mm (E, F). **G) **Myoblasts adhere to mantle epithelia (e). Asterisks: contractile material. Scale bar = 1.2 μm. **H) **Stage 8; phalloidin -FITC evidences thin mantle fibres (arrows) and muscle bundles in the adult (arrowheads). Scale bar = 0.1 mm. **I) **Two zooids approaching the opening of oral siphons (os). Arrows: muscle fibres. Scale bar = 0.1 mm. **J, K) **Zooids during regression. A decrease (compare Figures 2D-H) in the expression level of *BsMA2 *(J) and *BsTnT-c *(K) is recognisable. Fibres (arrows) assume an irregular aspect. Testis (ts; J) is not marked. (os), oral siphon; (en), endostyle. Scale bar = 0.1 mm. **L) **During regression, muscle fibres (mf)form cytoplasmic protrusions. Arrows: organelles in degeneration. Scale bar = 0.8 μm. **M) **The heart (arrow) of bud gives a signal only with *BsMA2*. (st), stomach. Scale bar = 25 μm. **N, O) **Early larvae. In the striated caudal muscles (arrows) no signal for *BsTnT-c *(N) and *BsMA2 *(O) expression. ISH of *BsMA2 *labels mesenchymal cells (arrowheads). Tunic is around ampullae (am) and cephalenteron and stains non-specifically. Scale bar = 50 μm.

We investigated the differentiation of unstriated muscles during the entire blastogenetic cycle, beginning from the appearance of a budlet to the adult stage and zooid regression. Results from phalloidin, TEM and ISH experiments allowed us to follow in detail the musculature modifications throughout each developmental stage (summarised in Figure 4A-L). Antisense RNA probes designed against *BsMA2 *and *BsTnT-c *mRNAs provided similar results.

In the early phases of bud development, no phalloidin signal was detected. Signal then began to be recognisable in the form of a diffuse fluorescence throughout the whole bud, but no discrete muscle fibres were distinguishable. At the same time, the first evidence of the diffuse expression of *BsMA2 *and *BsTnT-c *transcripts appeared just before the formation of a new blastogenetic generation from the body-wall of bud (stage 6). Both probes produce a diffuse signal, labelling a localised region between oral and atrial siphon and staining cells probably representing mesenchymal cells differentiating into myoblasts (Figures 4A-B). These cells begin to organise to form bundles of fibres; the siphons are in form of rudiments as epidermal thickenings. In the next stage, the body-wall musculature generates a network of fibres (Figure 4C) and signs of gene expression are observable in fibres that run along the dorsal lamina and through the recto-oesophageal trabecula (Figures 4D-F). The presence of differentiating myoblasts has been confirmed with TEM: they appear as circulating cells, which adhere and group to the mantle epithelia close to the developing siphons and are characterised by contractile material in their cytoplasm (Figure 4G).

Subsequently, the heart begins to beat and organogenesis progresses. Thin, discrete, apparently disorganised muscle fibres start to be recognisable with phalloidin (Figure 4H) and, approaching the opening of the siphon, the muscle fibres become thicker and reach the definitive regular organisation, extending laterally to envelope the rest of the body (Figure 4I). At this stage, the fibres that run around the velum and the pericoronal bands are particularly evident, as seen on ISH sections using both *BsMA2 *and *BsTnT-c *probes (not shown).

During takeover, the primary buds open their siphons, replacing the adult zooids, which are contracting to the centre of the system and are gradually reabsorbed. The regressing muscle fibres become more irregular and show decreased level in expression for both *BsMA2 *and *BsTnT-c *transcripts, as compared to the filtering zooids (Figures 4J-K). The contraction of fibres leads to the formation of cytoplasmic protrusions along the contractile material, which contain degenerating organelles (Figure 4L). The contractile material condenses to form a rigid structure, the paracrystalline body [66]. As with the other zooid tissues, the muscle cells are phagocytised by macrophages and digested in intracellular vacuoles.

#### Heart

Our observations agree with a previous report on heart development [65]. Briefly, in the buds of *B. schlosseri *the heart originates from a ventral vesicle, which forms from mesenchymal cells that invaginate along the dorsal lamina to form the myocardium (stage 4). The first regular heart beat characterises the transition of bud to stage 8, from which point a strong signal of *BsMA2 *expression is first recognisable (Figure 4M), limited to the cytoplasm of cardiac cells in the developing myocardium. This is maintained until zooid regression.

### *In situ *hybridisation in larval muscle

Antisense RNA probes for *BsMA2 *and *BsTnT-c *were tested in late embryos and larvae in order to verify if the two genes were expressed in musculature also during embryogenesis. Sections containing both the cephalenteron and tail of specimens at different stage show that transcripts of *BsTnT-c *are never detectable (Figure 4N). Also transcripts of *BsMA2 *are not detectable at the level of the caudal striated muscle. However, the *BsMA2 *probe gives strong signal at the oral siphon level where it labels mesenchymal cells, some of them dispersed in the branchial area, but most aggregated around the ectoderm of the rudiment of oral siphon (Figure 4O).

## Discussion

Previous research has shown that the unstriated muscle of adult ascidians possesses intermediate features between the striated and smooth muscles of vertebrates; it has multinucleate cells [17] and a contractile regulatory system based on the Tn/Tm apparatus similar to vertebrate striated muscle [15,16], but also lacks sarcomeric periodicity and regular spatial organisation of myofilaments, similar to vertebrate smooth muscle. From a developmental point of view, it is interesting to note that unstriated muscle differentiation in ascidians is associated with the formation of sessile zooids that occurs through metamorphic events, in the oozooid, or asexual reproduction in all the blastozooids.

During their life cycle ascidians also develop striated muscle in the tail of the free-swimming larvae and in the myocardium of adults. This has typical characteristics of the vertebrate striated muscles, such as the arrangement and the ratio of the myofilaments in the myofibrils (see [13,67]). The study of the different musculatures during the complete cycle of a colonial ascidian can be of interest from developmental and evolutionary perspectives, especially considering that the tunicates are the closest group (taxon) to vertebrates [68]. In this light, our study on *B. schlosseri *muscle allows us to better analyse the development and organisation of this system. In particular, we aimed to characterise two main genes (actin and TnT), whose expression patterns were investigated in the larva and during the life cycle of the blastozooids forming the colony. Moreover, our data contribute to a better understanding of the evolution and maintenance of the different muscle types in the chordate group.

### Actins

Our analyses confirm that actin genes are extremely well conserved in metazoans, with most variability confined to N-terminal region, that remains the most distinct feature amongst different actin isoforms and is exposed on the surface of the protein monomers [69]. CAs and MAs are distinguishable by the presence of residues at specific positions, and by a series of acidic amino acids involved in the interaction with myosin in the muscular actins [70].

Some ascidians, such as *C. intestinalis *[34] and *Molgula oculata *[GenBank: AAC28358, AAC28356], are known to possess different CA genes; accordingly, it is possible that *B. schlosseri *possesses other genes coding for CAs.

As demonstrated by our ISH experiments, *BsMA2 *is expressed in both unstriated and cardiac musculature of *B. schlosseri *blastozooids and in the presumptive adult muscle, but not in the striated musculature of the larva.

The alignment of the amino acid sequences shows that ascidian larval and adult MAs are very similar to the MAs of vertebrates. The two different ascidian MAs are well resolved from each other and belong to a well-defined group of chordate MAs. This suggests there was a single common ancestral gene in the chordate common ancestor from which evolved all the different MAs of the phylum.

The intron pattern of *BsMA2 *could be found in other ascidian MA2s and the position of the first intron seems to be conserved from vertebrate to echinoderm actin genes, apart for ascidian larval and amphioxus muscle forms (additional file 2). All the examined tunicate muscle actins do not exhibit the specific intron at position 328/329 which characterises the vertebrate forms, and it could be hypothesised that this intron was acquired during vertebrate evolution.

Our analysis on actin intron-exon organisation fits Kusakabe and collaborators' [35] depiction of a conserved deuterostome actin intron pattern, some of which are also present in protostomes (which have between zero and seven introns).

The intron positions of *BsCA1 *share the same pattern found in other ascidian CAs, such as *HrCA1 *(additional file 2). Two ascidian introns (150 and 204) are shared with the vertebrate muscle actins, but not with the cytoplasmic actins.

### Troponin T

The most probable scenario pictured by our analysis, considering animal phylogeny [68,71,72], predicts an ancestral TnT gene in the bilaterian common ancestor from which protostome and deuterostome TnTs evolved. In the deuterostome lineage, ascidian and vertebrate TnT isoforms evolved separately from an ancestral chordate TnT gene. The troponin T genes of vertebrates are normally involved in several splicing phenomena that result in a number of isoforms characterising different tissues or developmental stages. Our phylogenetic analysis confirms that the vertebrate TnT gene family arose by gene duplications within the vertebrate lineage and, at least in vertebrates, there is a similarity between TnT isoforms of the same muscle type in different species greater than between isoforms of different muscle types in the same species [34,73]. This "tissue-specific TnT isoforms homology" hypothesis could be also extended to ascidians as *BsTnT-c *is closely related to the adult isoform of *H. roretzi*, but more information is needed from other tunicate species to confirm this.

It has been shown that one TnT gene encodes for a specific larval and another for a body-wall muscle form in *H. roretzi*, while in *C. intestinalis *one gene codes for three isoforms, which are expressed respectively in the larva, heart and adult body-wall muscle [34,60]. In our study, we found that the expression of the *BsTnT-c *transcript is detected only in the body-wall musculature of zooids, without any signal at the myocardial level. We hypothesise that *B. schlosseri *possesses a different isoform expressed only in the heart muscle and probably the same situation is present in *H. roretzi*, in which only a form was isolated from the body-wall muscle [60].

### General Morphology

The study with phalloidin was successful in revealing the general organisation of the body-wall muscle in the blastozooids of *B. schlosseri *and, interestingly, a strict correspondence was found between the time of appearance and morphology of the muscle and the expression of the two investigated genes.

The muscle system of *B. schlosseri *follows the general organisation reported in other ascidians (see [67]). As expected, muscles concentrate in the mantle around the siphons where they are organised in a net of circular and longitudinal bands that are extended mainly along the dorso-lateral mantle. No muscle was observed in the branchia and gut, where food movement is caused by ciliary beating [74]. However, two bands run along the dorsal lamina penetrating the recto-oesophageal trabecula to reach the atrial siphon. They take part in the general contraction of the body. Tentacles of the oral siphon lack musculature and their movement depends on blood pressure. They expose the coronal organ with mechanosensory cells participating in the regulation of muscle activity to control the water flow through the body [63,75]. The general layout of the muscle innervation, described in detail in *B. schlosseri *buds [76], has shown that motor axons run out from cell bodies in the brain to the developing muscles of the zooid. However, excitation can extend to contiguous zooids and it was proposed that coordination of cardiac and body-wall muscle contractions in the zooids of the same colony can be achieved by electric impulses propagated through epithelial cells lining the body and the blood vessels in the tunic [77]. Heart beating begins early in the bud, before differentiation of the other organs [65] and continues also during take-over and regression of organs, in order to guarantee blood circulation for the distribution of tissue residuals in the entire colony.

With our original protocol for ISH on sections of *B. schlosseri *colonies and larvae, we recognised the structures expressing *BsMA2 *and *BsTnT-c*. At the same time these structures were analysed by means of cytochemical and ultrastructural techniques. This integrated study on muscle organisation allowed us to characterise the series of events for muscle appearance, differentiation and regression during the entire blastogenetic cycle and to recognise several critical differences between unstriated muscle and striated cardiac and larval musculature. During differentiation, we found that the mRNAs for *BsMA2 *and *BsTnT-c *were synthesised by cells a short time before their translation into proteins and that the first myoblasts could be revealed by different techniques (fluorescence, TEM, ISH) in the mantle around the rudiment of siphons. Phalloidin gave a diffuse light signal, whereas TEM recognised differentiating myoblasts among mesenchymal cells adhering to epithelia, thanks to the appearance of aggregating myofilaments in their cytoplasm. This recalls what Sugino et al. [78] observed in the siphons of the buds of another stolidobranch, *Simplegma reptans*. We showed that, in *B. schlosseri*, the probes for *BsMA2 *and *BsTnT-c *stained earlier and exactly the differentiating cells, in agreement with the idea that the probes specifically localise expressed mRNAs, whereas TEM and phalloidin recognise actin after polymerisation. Thus, the signal of phalloidin marks specifically the muscle only when a great number of thin filaments form fibres. Differently, when the zooids approached regression and shrank, muscle activity (with the exception of the heart) decreased markedly and stopped. In correspondence to these events, as previously reported by Burighel and Schiavinato [66], we showed that the disorganisation of muscle was accompanied by morphological alteration of cells with formation of cytoplasmic protrusions containing organelles. At the same time the two investigated muscle genes underwent a progressive reduction of transcript levels, as revealed by the decrease of the intensity of their expression signal with ISH.

Notably, both the probes do not label the striated muscle of the larva. This could suggest that different isoforms were expressed in the larval caudal muscle. However, a strong signal was recognised in the larva, due to the differentiating unstriated muscle in the rudiment of the siphon. In the solitary ascidian *C. intestinalis *no signal at this level was detected possibly due to a heterochronic difference in siphon development, because the embryonic life of *C. intestinalis *extends for 18 h, while the *B. schlosseri *embryo develops for 5 days in the atrial cavity of mother, thus its larva has more developed adult structures [67].

In cardiac, striated in the larval tail and unstriated in the adult body-wall muscle types of *B. schlosseri *we have found, respectively, three different combinations of the MA and TnT expression: BsMA2^+^/BsTnT-c^-^, BsMA2^-^/BsTnT-c^- ^and BsMA2^+^/BsTnT-c^+^. This indicates that the three ascidian muscle types differ not only "ultrastructurally", but also in the combinations of the subtype of the molecules that compose them.

## Conclusion

Common and divergent pathways in alternative developmental processes are an intriguing issue in evolutionary and developmental biology research (see [38] for review in ascidians), and this work adds an element in this field as our data indicate a putative common pathway between embryogenesis and blastogenesis in unstriated adult body-wall muscle of ascidians, which also displays a similar organisation in both solitary and colonial ascidians [see also [67]].

Our data agree with the idea that the smooth muscle of vertebrates does not derive directly from the unstriated muscle of ascidians, but from a striated muscle possessed by the ancestor of vertebrates [24]. Indeed, the body-wall muscle of ascidians possesses intermediate aspects between striated (e.g. multinucleate fibres, a troponin regulatory system and expression of MyoD-like transcription factors, nicotinic-type acetylcholine receptors-cholinergic) and smooth (absence of sarcomeric organisation) muscle of vertebrates [[10,20] and [23]]. This supports the idea that it derived from the striated muscle of the chordate ancestor, as an adaptation to the sessile life-style which requires slow contractions for fine regulation of water flow throughout the body and extended, rapid retraction (till 50% of length) of the body for defensive activities [24,67].

Tunicates show high muscle plasticity in that, besides the three kinds of muscles described in ascidians, they can also form other muscle types. Among thaliaceans (except for pyrosomids that possess an unstriated muscle similar to body-wall muscle of ascidians), doliolids and salps show unusual muscle bands encircling their bodies. Both taxa lack a T system, but salps have multinucleated striated muscle endowed with an extensive sarcoplasmic reticulum [79,80], whereas doliolids, capable of extremely rapid movements, possess obliquely striated muscle with no or scarce sarcoplasmic reticulum [81].

Moreover, the appendicularian *Oikopleura longicauda *has a typical caudal striated muscle with specific characters: muscle cell differentiation proceeds throughout a first mononucleated phase, (recalling the definitive larval muscle of ascidians), then followed by division of the nuclei and formation of a multinucleated musculature, in contrast with cell fusion that typically characterises vertebrate skeletal muscle [82,83]. In addition, at the molecular level, the actin sequence of appendicularians possesses intermediate features between the two ascidian types [82].

The characteristics and plasticity of tunicate muscle, possibly derived from an original striated muscle, together with the position in phylogenetic tree of actin and troponin ascidian genes, agree with the recent view that not cephalochordates, but ascidians are the sister group of vertebrates [68]. At the same time, in the light of the actual debate on the sessile or motile nature of chordate ancestor [84], all the above considerations agree with the idea that a motile animal possessing the genetic machinery for differentiation of the typical chordate striated muscle was the common chordate ancestor, from which the tunicates originated and evolved their variety of muscles.

## Authors' contributions

VD and FG designed the study and drafted the manuscript; VD performed the analysis of genomic sequences; VD, FG, CS identified, analysed and cloned transcripts, and performed ISH; SMS, PB and LM participated in the design of the study and helped draft the manuscript, SMS and FG conducted the phylogenetic analyses; PB and LM performed histochemistry, ultrastructure and helped with analysis and interpretations of ISH. All authors read and approved the final manuscript.

## Supplementary Material

Additional file 1 **Figure S1**. Table reassuming the primers used in PCR reactions, as described in the text. Click here for file

Additional file 2 **Figure S2**. Intron positions in protein-coding region of *BsMA2 *and *BsCA1 *and actin genes of other organisms. Click here for file

Additional file 3 **Figure S3**. Molecular phylogenetic analysis of the actin protein family. Click here for file

Additional file 4 **Figure S4**. Comparison at specific amino acid positions of actins from different organisms. Click here for file

Additional file 5 **Figure S5**. Alignment of 65 complete amino acid sequences of muscle and cytoplasmatic actins from metazoans. Click here for file

Additional file 6 **Figure S6**. The N-termini of cytoplasmic and muscle actins of various species. Click here for file

Additional file 7 **Figure S7**. Alignment of troponin T sequences. Click here for file

Additional file 8 **Figure S8**. Pairwise scores calculated between TnTs of some deuterostomes compared to a rabbit fast skeletal muscle form. Click here for file
