# Supplementary material for: Muscle differentiation in a colonial ascidian: organisation, gene expression and evolutionary considerations
Source: BMC Dev Biol. 2009 Sep 8;9:48. doi: 10.1186/1471-213X-9-48 (PMC2753633; doi:10.1186/1471-213X-9-48)
Supplement: Additional file 1 — Figure S1. Table reassuming the primers used in PCR reactions, as described in the text. [file 1471-213X-9-48-S1.pdf]

***BsCA1* mRNA**	pDNR-Lib-FW1	5'-TTATCAGTCGACGGTACCGGACAT-3'
	BsCA-RW1	5'-AGGCGACATTAAGAGCTGCAGGAA-3'
***BsTnT-c* mRNA**	BsTnT-FW1	5'-AGAACAGGAAGCTCCACAAGAGGT-3'
	BsTnT-FW2	5'-AGGAATATGAACAGCAGGAGCAGG-3'
	pDNR-Lib-RW1	5'-CGCCAAACGAATGGTCTAGAAAGC-3'
***BsMA2* genomic**	MAgen_R	5'-ATGGAACAGGACGATGAAG-3'
	MAgen_F	5'-GTGGACAATGGAAGGACC-3'
***BsCA1* genomic**	5'BsCA-gen	5'-ATCAAGATGTGTGACGACG-3'
	3'BsCA-gen	5'- CGCAGTTTACAGGTTCAGGTCTA-3'

Figure S1. Primers used in PCR reactions as described in the text.
