# Supplementary material for: Muscle differentiation in a colonial ascidian: organisation, gene expression and evolutionary considerations
Source: BMC Dev Biol. 2009 Sep 8;9:48. doi: 10.1186/1471-213X-9-48 (PMC2753633; doi:10.1186/1471-213X-9-48)
Supplement: Additional file 2 — Figure S2. Intron positions in protein-coding region of BsMA2 and BsCA1 and actin genes of other organisms. [file 1471-213X-9-48-S2.pdf]

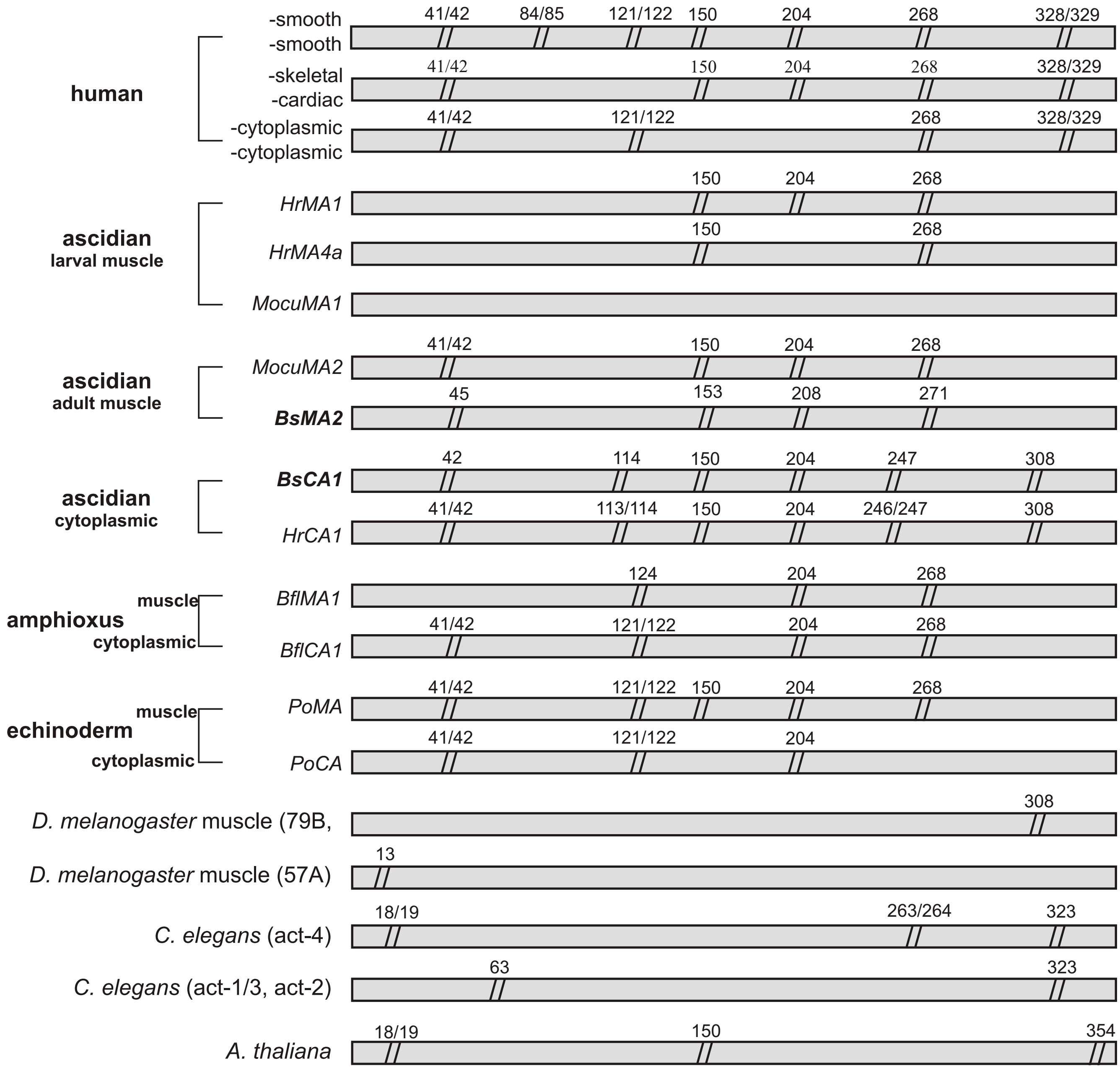

Figure S2. Intron positions in protein-coding region of *BsMA2* and *BsCA1* and actin genes of other organisms.

BsMA2 (bold) shows four introns and their positions are conserved in all muscle actins of deuterostomes, with the exception of the larval muscle forms of ascidian [28-30]. In *BsCA1* (bold) six introns are present. The horizontal grey boxes represent the amino acid sequence with the insertion sites of the introns (oblique bars). Actin sequences and their accession numbers included in the comparison are: human -cardiac, -skeletal, -smooth, -cytoplasmic, -cytoplasmic and -smooth (*Homo sapiens*; J00073, M20543, X13839, M10277, M19283 and X16940) *HrMA1*, *HrMA4a* and *HrCA1* (*H. roretzi*; D29014, D10887 and D45164), *MocuMA1* and *MocuMA2* (*Molgula oculata*; D78190 and D85743), *BsMA2* and *BsCA1* (*B. schlosseri*, see text for accession numbers), *BflMA1* and *BflCA1* (*B. floridae*; fgenesh2_kg.scaffold_259000003(*) and fgenesh2_kg.scaffold_218000001(*)), *PoMA* and *PoCA* (*Pisaster ochraceus*; M26500 and M26501), *Act79B/88F* and *Act57A* (D. melanogaster; CG7478(°) and CG10067(°)), *act-4*, *act-1/3* and *act-2* (*Caenorhabditis elegans*; M03F4.2(+), T04C12.4(+) and T04C12.5(+)) and *A. thaliana* (*Arabidopsis thaliana*; M20016). (*), (°) and (+) are downloaded from JGI, FlyBase and WormBase database respectively, all the other ones are from GenBank.
