# Supplementary material for: Muscle differentiation in a colonial ascidian: organisation, gene expression and evolutionary considerations
Source: BMC Dev Biol. 2009 Sep 8;9:48. doi: 10.1186/1471-213X-9-48 (PMC2753633; doi:10.1186/1471-213X-9-48)
Supplement: Additional file 3 — Figure S3. Molecular phylogenetic analysis of the actin protein family. [file 1471-213X-9-48-S3.pdf]

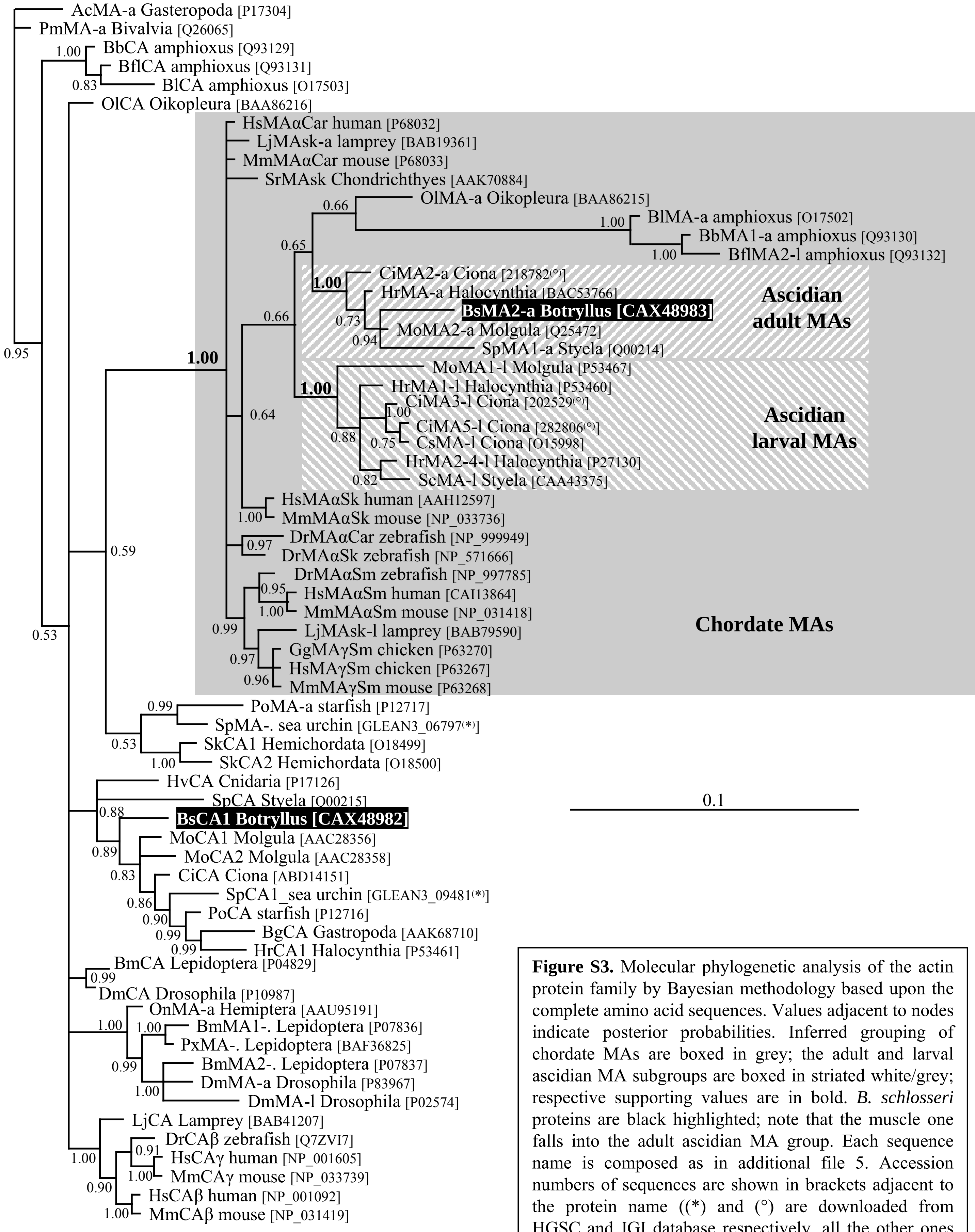

Figure S3. Molecular phylogenetic analysis of the actin protein family by Bayesian methodology based upon the complete amino acid sequences. Values adjacent to nodes indicate posterior probabilities. Inferred grouping of chordate MAs are boxed in grey; the adult and larval ascidian MA subgroups are boxed in striated white/grey; respective supporting values are in bold. *B. schlosseri* proteins are black highlighted; note that the muscle one falls into the adult ascidian MA group. Each sequence name is composed as in additional file 5. Accession numbers of sequences are shown in brackets adjacent to the protein name ((*) and (°) are downloaded from HGSC and JGI database respectively, all the other ones form GenBank).
