# Supplementary material for: Muscle differentiation in a colonial ascidian: organisation, gene expression and evolutionary considerations
Source: BMC Dev Biol. 2009 Sep 8;9:48. doi: 10.1186/1471-213X-9-48 (PMC2753633; doi:10.1186/1471-213X-9-48)
Supplement: Additional file 4 — Figure S4. Comparison at specific amino acid positions of actins from different organisms. [file 1471-213X-9-48-S4.pdf]

	Amino acid position																			
	5	6	10	16	17	76	103	129	153	162	176	201	225	260	267	272	279	287	297	365
Human α-skeletal	T	T	C	L	V	I	T	V	L	N	M	V	N	T	I	A	Y	I	N	A
Human α-cardiac	T	T	C	L	V	I	T	V	L	N	M	V	N	T	I	A	Y	I	N	A
Human α-smooth	S	T	C	L	C	I	T	V	L	N	M	V	N	T	I	A	Y	I	N	A
Human γ-smooth	T	T	C	L	C	I	T	V	L	N	M	V	N	T	I	A	Y	I	N	A
**Ascidian *BsMA2-a***	**Q**	**T**	**C**	**L**	**V**	**I**	**V**	**V**	**F**	**N**	**M**	**V**	**Q**	**T**	**V**	**S**	**Y**	**I**	**N**	**S**
Ascidian *CiMA2-a*	Q	T	C	L	V	I	V	V	L	N	M	V	Q	T	I	S	Y	I	N	A
Ascidian *HrMA2/4-l*	T	T	C	L	V	I	T	V	L	N	A	V	Q	T	I	A	Y	I	N	A
Ascidian *CiMA5-l*	Q	T	C	L	V	I	T	V	M	N	A	V	Q	T	I	A	Y	I	N	A
Appendicularian *OlMA-a*	Q	T	C	L	V	I	T	V	L	N	M	V	Q	T	I	A	Y	I	N	A
Amphioxus *BlMA-a*	A	T	C	L	V	V	C	S	L	T	L	V	Q	A	L	A	Y	I	N	A
Starfish *PoMA-a*	V	A	V	M	C	V	V	S	F	T	L	T	Q	T	I	A	Y	I	T	S
Drosophila *DmMA-a*	A	G	I	M	C	I	V	S	L	T	L	T	Q	A	L	C	Y	V	S	S
Human β-cytoplasmic	I	A	V	M	C	V	V	T	M	T	L	T	Q	A	L	C	F	V	T	S
Human γ-cytoplasmic	I	A	I	M	C	V	V	T	M	T	L	T	Q	A	L	C	F	V	T	S
**Ascidian *BsCA1***	**S**	**A**	**V**	**M**	**C**	**V**	**V**	**T**	**F**	**T**	**L**	**T**	**Q**	**A**	**L**	**A**	**Y**	**V**	**T**	**S**
Ascidian *CiCA*	V	A	V	M	C	V	V	T	F	T	L	T	Q	A	L	A	Y	V	T	S
Appendicularian *OlCA*	V	A	V	M	C	V	V	T	L	T	L	T	Q	A	L	A	Y	V	T	S
Amphioxus *BlCA*	V	A	V	M	C	V	L	S	L	T	L	T	Q	S	L	T	Y	I	T	S
Starfish *PoCA*	V	A	V	M	C	V	V	T	F	T	L	T	Q	A	L	A	Y	V	T	S
Drosophila *DmCA*	V	A	V	M	C	V	V	T	L	T	L	T	Q	A	L	C	Y	V	T	S

Figure S4. Comparison at specific amino acid positions of actins from different organisms.

The positions shown were chosen on the basis of diagnostic differences between mammalian skeletal α- actin and cytoplasmatic β-actin. The upper group contains muscle actins, while below cytoplasmic actins are listed. The position numbers of the residues are based on human skeletal α-actin, in which position number 1 is a D encoded by the third codon [85] (see below for the reference). Shared residues are in grey. The same sequences listed in this table are used for phylogenetic analysis and are shown in full in the alignment in additional file 5.

85. Vandekerckhove J, Weber K: **Chordate muscle actins differ distinctly from invertebrate muscle actins. The evolution of the different vertebrate muscle actins.** *J Mol Biol* 1984, **179:**391-413.
