# Supplementary material for: Muscle differentiation in a colonial ascidian: organisation, gene expression and evolutionary considerations
Source: BMC Dev Biol. 2009 Sep 8;9:48. doi: 10.1186/1471-213X-9-48 (PMC2753633; doi:10.1186/1471-213X-9-48)
Supplement: Additional file 5 — Figure S5. Alignment of 65 complete amino acid sequences of muscle and cytoplasmatic actins from metazoans. [file 1471-213X-9-48-S5.pdf]

Figure S5 (p 1/4)

```
                                    10        20        30        40        50        60        70        80        90       100       110       120       130
                            ....|....|....|....|....|....|....|....|....|....|....|....|....|....|....|....|....|....|....|....|....|....|....|....|....|....|
HrMA-a_Halocynthia          -ME-DEEEEQTALVCDNGSGLVKAGFAGDDAPRAVFPSIVGRPRHQGVMVGMGQKDSYVGDEAQSKRGILTLKYPIEHGIITNWDDMEKIWHHTFYNELRVAPEEHPVLLTEAPLNPKANREKMTQIMFE
MoMA2-a_molgula             -..-..............................................................................................................................
CiMA2-a_Ciona               -.S-E.............................................................................................................................
BsMA2-a_Botryllus           -..Q.D............................................................................................................................
SpMA1-a_Styela              -..D.QD...................P..AP......LT..............................................N............................................
HrMA2/4-l_Halocynthia       --MS.G..DT..I..........S...................................................................................T......................
ScMA-l_Styela               --MS.G..D...I..........S......................................................................M............T......H...............
HrMA1-l_Halocynthia         --MS.G..D...I..........S..............T....................................................................T......................
CsMA-l_Ciona                --MS.S..D..............S...................................................................................T......................
CiMA5-l_Ciona               --MS.S..D..............S...................................................................................T......................
CiMA3-l_Ciona               --MS.S..D..............S...................................................................................T......................
MoMA1-l_Molgula             --MSS...D...I.......................................................................................I......T......................
OlMA-a_Oikopleura           -.DSE..............................Y.................S...................................V..........I......T......................
DrMAαCar_zebrafish          ---MCDDD.T.................................................................................................T......................
DrMAαSk_zebrafish           ---MCDD..T.................................................................................................T......................
HsMAαSk_human               ---MCD.D.T.................................................................................................T......................
MmMAαSk_mouse               ---MCD.D.T.................................................................................................T......................
LjMAsk-a_lamprey            ---MCDD..T...........C.....................................................................................T......................
HsMAαCar_human              ---MCDD..T.................................................................................................T......................
MmMAαCar_mouse              ---MCDD..T.................................................................................................T......................
SrMAsk_Chondrichthyes       ---MCDD..T..............................................A..................................................T...................L..
HsMAαSm_human               ---MC...DS...........C.......................................................................S.............T......................
MmMAαSm_mouse               ---MC...DS...........C.......................................................................S.............T......................
DrMAαSm_zebrafish           ---MCDD..S...........C.......................................................................S.............T......................
GgMAγSm_chicken             ---MC-...T...........C.......................................................................S.............T......................
HsMAγSm_human               ---MC-...T...........C.......................................................................S.............T......................
MmMAγSm_mouse               ---MC-...T...........C.......................................................................S.............T......................
LjMAsk-l_lamprey            ---MC-D..T...........C.......................................................................S......I......T......................
SpMA-._sea-urchin           ----MCD.DVA...V.....MC..........................................................V.................................................
PoMA-a_starfish             ----MCD.DVA...V.....MC..........................................................V.................................................
SkCA1_Hemichordata          ----MCD..VA...V.....MC............I.............................................V.................................................
SkCA2_Hemichordata          ----MCD..VA...V.....MC..........................................................V.................................................
AcMA-a_Gasteropoda          ----MCDD.VA...V.....MC..........................................................V....................P............................
PmMA-a_Bivalvia             ----MCDD.VA...V.....MC..........................................................V.................................................
BgCA_Gastropoda             ----MCD.DVA...V.....MC..........................................................V.................................................
HrCA1_Halocynthia           ----MCD.DVA...V.....MC..........................................................V.................................................
PoCA_starfish               ----MCD.DVA...V.....MC..........................................................V.................................................
CiCA_Ciona                  -----MDDDVA...V.....MC..........................................................V.................................................
SpCA1_sea-urchin            ----MCDDDVA...I.....M...........................................................V.................................................
MoCA1_Molgula               -----MDDDVA...V.....MC..........................................................V.................................................
MoCA2_Molgula               -----MDDDVA...V.....MC..........................................................V.................................................
BsCA1_Botryllus             ----MCDD.SA...V.....MC..........................................................V.................................................
SpCA_Styela                 -----MDD.VA...V.....MC.....E........................V...........................V.................................................
HvCA_Cnidaria               ----MADD.VA...V.....MC..........................................................V.................................................
BmCA_Lepidoptera            ----MCD..VA...V.....MC..........................................................V.................................................
DmCA_Drosophila             ----MCD..VA...V.....MC..........................................................V.................................................
OlCA_Oikopleura             -----MDD.VA...V.....MC..........................................................V.................................................
HsCAγ_human                 -----M...IA...I.....MC..........................................................V.................................................
MmCAγ_mouse                 -----M...IA...I.....MC..........................................................V.................................................
DrCAβ_zebrafish             -----MD..IA...V.....MC..........................................................V.................................................
HsCAβ_human                 -----MDDDIA...V.....MC..........................................................V.................................................
MmCAβ_mouse                 -----MDDDIA...V.....MC..........................................................V.................................................
LjCA_lamprey                ----MA.D.IA...V.....MC..........................................................V.................................................
PxMA-._Lepidoptera          ----MCDDDVA...V.....MC............................................................................................................
BmMA1-._Lepidoptera         ----MCDDDVR...V.....MC.........................L..................................................................................
BmMA2-._Lepidoptera         ----MCDDDAG...V.....MC.....................................................................................I......................
DmMA-a_Drosophila           ----MCDDDAG...I.....MC............................................................................................................
DmMA-l_Drosophila           ----MCD..AS...V.....MC..................................C.............S..................V........................................
OnMA-a_Hemiptera            ----MCDDDVA...V.....MC.....................................................................................I......................
BbCA_amphioxus              -----M.DDVA...V.....MC..........................................................V.................................................
BflCA_amphioxus             -----M.DDVA...V.....MC..........................................................V.................................................
BlCA_amphioxus              -----MDDDVA...V.....MC.....................................................V....V...................I....N.L......................
BbMA1-a_amphioxus           MCDDE...-A.P............................................A..................V....V........V..........I....N.C......................
BflMA2-l_amphioxus          -.SDE...-A.P............................................A..................V....V........V..........I....N.C......................
BlMA-a_amphioxus            MCDDE....A.P............................................A..................V....V...................I....N.C......................
Clustal Consensus                 ::   .:* *****: *:**. * .***::*  ********:**** .**.*************:****:****:****:***:***::*****:.***:* ****** ************:**
```

```
Figure S5 (p 2/4)                    140       150       160       170       180       190       200       210       220       230       240       250       260
                             ....|....|....|....|....|....|....|....|....|....|....|....|....|....|....|....|....|....|....|....|....|....|....|....|....|....|
HrMA-a_Halocynthia           TFNVPAMYVAIQAVLSLYASGRTTGIVLDSGDGVSHNVPIYEGYALPHAIMRLDLAGRDLTDYLMKILTERGYSFVTTAEREIVRDIKEKLCYVALDFEQEMATAASSSSLEKSYELPDGQVITIGNERF
MoMA2-a_molgula              ..................................................................................................................................
CiMA2-a_Ciona                .............................................................................................................G....................
BsMA2-a_Botryllus            ...........................F..................................................GK..................................................
SpMA1-a_Styela               ..................................................................................................................................
HrMA2/4-l_Halocynthia        .............................A....................A.........................................................T.....................
ScMA-l_Styela                ...........................M.A....................A.........................................................T.....................
HrMA1-l_Halocynthia          ..................................................A.........................................................T.....................
CsMA-l_Ciona                 ...........................M.A....................A.........................................................T.....................
CiMA5-l_Ciona                ...........................M.A....................A.........................................................T.....................
CiMA3-l_Ciona                ...........................M......................A.........................................................T.....................
MoMA1-l_Molgula              .................................I................A.M........E..T...............K...........................T.....................
OlMA-a_Oikopleura            .........C...................A.........V..........................................................................................
DrMAαCar_zebrafish           ...................................................................................................N...........G..................
DrMAαSk_zebrafish            .............................A....T....V...........................................................N..............................
HsMAαSk_human                ..................................T................................................................N..............................
MmMAαSk_mouse                ..................................T................................................................N..............................
LjMAsk-a_lamprey             ..................................T................................................................N......T.......................
HsMAαCar_human               ..................................T................................................................N..............................
MmMAαCar_mouse               ..................................T................................................................N..............................
SrMAsk_Chondrichthyes        ..................................T................................................................N..............................
HsMAαSm_human                ..................................T................................................................N..............................
MmMAαSm_mouse                ..................................T................................................................N..............................
DrMAαSm_zebrafish            ..................................T................................................................N......F.......................
GgMAγSm_chicken              ..................................T................................................................N..............................
HsMAγSm_human                ..................................T................................................................N..............................
MmMAγSm_mouse                ..................................T................................................................N..............................
LjMAsk-l_lamprey             ..................................T................................................................N..TM..........................
SpMA-._sea-urchin            ...A..............................T.T.............L........................T.................T............A.......................
PoMA-a_starfish              ...S.......................F.T......T.............L........................T.................T........Q..S........................
SkCA1_Hemichordata           ...T.......................M.T....T.T.............L........................T......................................................
SkCA2_Hemichordata           ...T.......................M.T....T.T.............L........................T......................................................
AcMA-a_Gasteropoda           ...A..............................T.T.............L.............S..........T......................................................
PmMA-a_Bivalvia              ...A..............................T.T.............L........................T.......................N..............................
BgCA_Gastropoda              ...T.......................M......T.T......................................T..........................Q..ST........................
HrCA1_Halocynthia            ...T.......................F........T.............L........................T...............A.......T..Q...T...I.............V.....
PoCA_starfish                ...T.......................F........T.............L........................T..........................Q...........................
CiCA_Ciona                   ...T.......................F........T.............L........................T..........................Q.....................V.....
SpCA1_sea-urchin             ...S.......................F........T.............L........................T..........................Q...........................
MoCA1_Molgula                ...T.......................F........T.............L........................T..........................S...........................
MoCA2_Molgula                ...C.......................F........T.............L......................A.T..................G.......G...........................
BsCA1_Botryllus              ...T.......................F........T.............L........................T..........................Q......T.............V.....
SpCA_Styela                  ...TR....N...............L.M........T.............F........................T..................G....S..G..S...V....................
HvCA_Cnidaria                ...S................................T.............I........................T...............S..........Q...........................
BmCA_Lepidoptera             ...T................................T.............L........................T......................................................
DmCA_Drosophila              ...T................................T.............L........................T......................................................
OlCA_Oikopleura              ...T................................T..........Y..L........................T......................................................
HsCAγ_human                  ...T.......................M......T.T.............L........................T......................................................
MmCAγ_mouse                  ...T.......................M......T.T.............L........................T......................................................
DrCAβ_zebrafish              ...T.......................M......T.T.............L........................T..........................G...........................
HsCAβ_human                  ...T.......................M......T.T.............L........................T......................................................
MmCAβ_mouse                  ...T.......................M......T.T.............L........................T......................................................
LjCA_lamprey                 ...T.......................M........T.............L........................T......................................................
PxMA-._Lepidoptera           ...S................................T.............L........................T..............................A.T.....................
BmMA1-._Lepidoptera          ...S................................T.............L........................T..............................A.T.....................
BmMA2-._Lepidoptera          ...C................................T.............L........................T..........................Q...A.T.....................
DmMA-a_Drosophila            ...S................................T......F.......L........................T..............................A.T.....................
DmMA-l_Drosophila            ...S................................T.............L........................T..............................A.T.....................
OnMA-a_Hemiptera             ...T................................T.............L........................T..............................A.T.....................
BbCA_amphioxus               ...S................................T.............L........................T......................................................
BflCA_amphioxus              ...S................................T.............L........................T..........................S...........................
BlCA_amphioxus               ...S................................T.............L........Q...............T..........................S...........................
BbMA1-a_amphioxus            ...S.....C.....C....................T........M....L.......E..N...RVMCD...A.................G..........L...T.T.....................
BflMA2-l_amphioxus           ...S.....C.....C....................T........M....L.......E..N...RVMCD...A.................G..........L...T.T.....................
BlMA-a_amphioxus             ...S.....C.....C....................T.............L.......E..N.....M.D.....................G..........L...T.T.....................
Clustal Consensus            ***  **** *****.*********:*:*:***::*.**:***:*:*:** *:*****: *:** ::: :***:*.**.::********** *..**** **  *: *: : ************:*****
```

Figure S5 (p 3/4)

```
                                    270       280       290       300       310       320       330       340       350       360       370       380
                            ....|....|....|....|....|....|....|....|....|....|....|....|....|....|....|....|....|....|....|....|....|....|....|....|
HrMA-a_Halocynthia          RCPETMFQPSFIGMESSGVHETTYNSIMKCDIDIRKDLYANNVLSGGTTMYPGIADRMQKEITALAPSTMKIKIIAPPERKYSVWIGGSILASLSTFQQMWITKQEYDEAGPSIVHRKCF
MoMA2-a_molgula             .............................................................................................................S..........
CiMA2-a_Ciona               ........................................................................................................................
BsMA2-a_Botryllus           ...........V.......................................................AP..........................P.............S..........
SpMA1-a_Styela              .....L.................................................................S...............A.....................S..........
HrMA2/4-l_Halocynthia       .....L..........A.I...................................................................................S.................
ScMA-l_Styela               .....L..........A.I...................................................................................S.................
HrMA1-l_Halocynthia         .....L..........A.I............................S........................................................................
CsMA-l_Ciona                .....L..........A.I.....................................................................................................
CiMA5-l_Ciona               .....L..........A.I.....................................................................................................
CiMA3-l_Ciona               .....L..........A.I.....................................................................................................
MoMA1-l_Molgula             .....L..........A.I........................................................................S.....N......A...............
OlMA-a_Oikopleura           .....L..........A.................................................................................A.....A...............
DrMAαCar_zebrafish          .....L..........A.I...A......................................................................P..........................
DrMAαSk_zebrafish           .....L..........A.I...A.................................................................................................
HsMAαSk_human               .....L..........A.I........................M............................................................................
MmMAαSk_mouse               .....L..........A.I........................M............................................................................
LjMAsk-a_lamprey            .....L..........A.I.....................................................................................................
HsMAαCar_human              .....L..........A.I...................................................................................S.................
MmMAαCar_mouse              .....L..........A.I...................................................................................S.................
SrMAsk_Chondrichthyes       .....L..........D.I...................................................................................S.................
HsMAαSm_human               .....L..........A.I...................................................................................S.................
MmMAαSm_mouse               .....L..........A.I...................................................................................S.................
DrMAαSm_zebrafish           .....L..........A.I...................................................................................S.................
GgMAγSm_chicken             .....L..........A.I...................................................................................S.P...............
HsMAγSm_human               .....L..........A.I...................................................................................S.P...............
MmMAγSm_mouse               .....L..........A.I...................................................................................S.P...............
LjMAsk-l_lamprey            .....L..........A.I................................................T..................................S.P...............
SpMA-._sea-urchin           .....L...A......A.I......................T......S...................S.................................S......S..........
PoMA-a_starfish             .....L...A......A.I......................T......S.............Q....P..................................S......S..........
SkCA1_Hemichordata          ....AL...A.L....P.I......................T......S..............S......................................S......S..........
SkCA2_Hemichordata          ....AL...A.L....P.I.....................FS......S..............S....S.................................S......S..........
AcMA-a_Gasteropoda          ....SL...ILL....A.I............V.........T........F............S......................................S......S..........
PmMA-a_Bivalvia             ....SL.....L....A.I............V.........T........F...................................................S......S..........
BgCA_Gastropoda             ....AT.....L...AA.I............V.........T.....S..F................P..................................S......S..........
HrCA1_Halocynthia           ....AL.....L....A.I............V.........T.....S..F...........V....P..................................S......S..........
PoCA_starfish               ....AL.....L....A.I............V.........T.....S..F..........V.....P..................................S......S..........
CiCA_Ciona                  ....AL.....L....A.I............V.........T.....S..F................P..................................S......S..........
SpCA1_sea-urchin            ....AL...A.L....A.I...C........V.........T.....S..F................P..................................S......S..........
MoCA1_Molgula               ....AL.....L....A.I...S........V.........T.....S...................P..................................S......S..........
MoCA2_Molgula               ....AL.....L....A.I............V.........T.....S...................P..................................S......S..........
BsCA1_Botryllus             ....AL.....L....A.I............V.........T.....S..............SN...P..................................S......S..........
SpCA_Styela                 ....A......L....A.I............V.........T....................S....P..................................S......S..........
HvCA_Cnidaria               .....L..........A.I............V.........T........F...........S....P..................................S......S..........
BmCA_Lepidoptera            ....AL.....L...AN.I............V.........T.....................R......................................S......S..........
DmCA_Drosophila             ....AL.....L...AC.I............V.........T............................................................S......S..........
OlCA_Oikopleura             ....AL.....L...AA.I............V.........T............................................................S......S..........
HsCAγ_human                 ....AL.....L....C.I....F.......V.........T............................................................S......S..........
MmCAγ_mouse                 ....AL.....L....C.I....F.......V.........T............................................................S......S..........
DrCAβ_zebrafish             ....AL.....L....C.I....F.......V.........T.....................S......................................S......S..........
HsCAβ_human                 ....AL.....L....C.I....F.......V.........T............................................................S......S..........
MmCAβ_mouse                 ....AL.....L....C.I....F.......V.........T............................................................S......S..........
LjCA_lamprey                ....AL.....L....C.I....F.......V.........T............................................................S......S..........
PxMA-._Lepidoptera          ....AL.....L....C.I...V........V.........T.M.........................I................................S.E....S..G.......
BmMA1-._Lepidoptera         ....AL.....L....C.I...V........V.........T.M.........................I................................S.E....S..G.......
BmMA2-._Lepidoptera         ....AL.....L....C.I...V........V.........T...........................I................................S......S..G.......
DmMA-a_Drosophila           ....AL.....L....C.I...V........V.........S...........................I................................S......S..G.......
DmMA-l_Drosophila           .T..AL.....L....C.I...V.Q......V.....................................I................................S......S..G.......
OnMA-a_Hemiptera            ....AL.....L....C.I...V........V.........T...........................I................................S......S..G.......
BbCA_amphioxus              ....SL.....L....T........................T........F...................................................S......S..........
BflCA_amphioxus             ....SL.....L....T........................T........F...................................................S......S..........
BlCA_amphioxus              ....SL.....L....T........................T........F...................................................S......S..........
BbMA1-a_amphioxus           ..A.AL.....L....A.....V..........V................F...G......MVS...................C.............AA...K.S.......A.......
BflMA2-l_amphioxus          ..A.AL.....L....A.....V..........V................F...G......MV....................C.............AS...K.S.......A.......
BlMA-a_amphioxus            ..A.AL.....L....A.....V..........V................F...G......MVS...................C.............AA...K.S.......A.......
Clustal Consensus           * .*: *** ::***: *:*** ::******:*:****** .*:***::*:***.******:  ***..:* *********** ***.***:* *.*  ***.* ****:**.*******
```

Figure S5. Alignment of 65 complete amino acid sequences of muscle and cytoplasmatic actins from metazoans, that illustrates theirs high similarity, as many sites are invariant. Diagnostic residues positions distinctive for the vertebrate muscle and cytoplasmic actin forms (see additional file 4) are boxed in grey. Each sequence name is composed as follow: an acronym of the specie, followed by i) the type of actin (MA: muscle actin; CA: cytoplasmic actin; α: alfa; β: beta; γ: gamma), ii) the type of muscle if known (Sk: skeletal muscle; Sm: smooth muscle; Car: cardiac), iii) the developmental stage if known (-a: adult; -l: larval; -.: unknown), iv) an ID name to recognise different species with same acronym. Acronym/ID name associations identify the following species: Ac/Gasteropoda, *Aplysia californica*; Bb/amphioxus, *Branchiostoma belcheri*; Bfl/amphioxus, *Branchiostoma floridae*; Bg/Gastropoda, *Biomphalaria glabrata*; Bl/amphioxus, *Branchiostoma lanceolatum*; Bm/Lepidoptera, *Bombyx mori*; Bs/Botryllus, *Botryllus schlosseri*; Ci/Ciona, *Ciona intestinalis*; Cs/Ciona, *Ciona savignyi*; Dm/Drosophila, *Drosophila melanogaster*; Dr/zebrafish, *Danio rerio*; Gg/chicken, *Gallus gallus*; Hr/Halocynthia, *Halocynthia roretzi*; Hs/human, *Homo sapiens*; Hv/Cnidaria, *Hydra vulgaris*; Lj/lamprey, *Lethenteron japonicum*; Mm/mouse, *Mus musculus*; Mo/Molgula, *Molgula oculata*; Ol/Oikopleura, *Oikopleura longicauda*; On/Hemiptera, *Oncometopia nigricans*; Pm/Bivalvia, *Placopecten magellanicus*; Po/starfish, *Pisaster ochraceus*; Px/Lepidoptera, *Plutella xylostella*; Sc/Styela, *Styela clava*; Sk/Hemichordata, *Saccoglossus kowalevskii*; Sp/sea urchin, *Strongylocentrotus purpuratus*; Sp/Styela, *Styela plicata*; Sr/Chondrichthyes, *Scyliorhinus retifer*. For the other accession numbers see additional file 3.
