# Supplementary material for: Muscle differentiation in a colonial ascidian: organisation, gene expression and evolutionary considerations
Source: BMC Dev Biol. 2009 Sep 8;9:48. doi: 10.1186/1471-213X-9-48 (PMC2753633; doi:10.1186/1471-213X-9-48)
Supplement: Additional file 6 — Figure S6. The N-termini of cytoplasmic and muscle actins of various species. [file 1471-213X-9-48-S6.pdf]

Human α-skeletal	--MCD-EDETTALVCDNGSGLVKA
Human α-cardiac	--MCD-DEETTALVCDNGSGLVKA
Human α-smooth	--MCE-EEDSTALVCDNGSGLCKA
Human γ-smooth	--MCE-EET-TALVCDNGSGLCKA
Ascidian *HrMA2/4*	--MSDGEEDTTAIVCDNGSGLVKS
Ascidian *CiMA5*	--MSDSEEDQTALVCDNGSGLVKS
Ascidian *CiMA2*	--MSEEEEEQTALVCDNGSGLVKA
**Ascidian *BsMA2***	**-MEQDDEEEQTALVCDNGSGLVKA**
Appendicularian *OlMA*	-MDSEEEEEQTALVCDNGSGLVKA
Amphioxus *BlMA*	MCDDEEEEEATPLVCDNGSGLVKA
Starfish *PoMA*	----MCDEDVAALVVDNGSGMCKA
Drosophila *DmMA*	----MCDDDAGALVIDNGSGMCKA
Human β-cytoplasmic	-----MDDDIAALVVDNGSGMCKA
Human γ-cytoplasmic	-----MEEEIAALVIDNGSGMCKA
Ascidian *CiCA*	-----MDDDVAALVVDNGSGMCKA
**Ascidian *BsCA1***	**----MCDDESAALVVDNGSGMCKA**
Appendicularian *OlCA*	-----MDDEVAALVVDNGSGMCKA
Amphioxus *BlCA*	-----MDDDVAALVVDNGSGMCKA
Starfish *PoCA*	----MCDEDVAALVVDNGSGMCKA
Drosophila *DmCA*	----MCDEEVAALVVDNGSGMCKA

Figure S6. The N-termini of cytoplasmic and muscle actins of various species.

BsMA2 (in bold) lacks the Cys residue (grey boxes) after the first Met, but possesses a series of six acidic amino acids (E and D, underlined). This is consistent with the situation of other chordates, where the muscle actins are characterised by at least four acidic residues in the first positions, probably involved in the interaction with the lysine-rich loop of the myosin head [70]. *BsCA1* (residues in bold) has a Cys next to the first Met like non-chordate cytoplasmic actins and lacks both the typical acidic amino acids and the residues that characterise the muscle isoforms (black boxes). Actin sequences included in the comparison: human actins, *HrMA2/4* (*Halocynthia roretzi*), *CiMA5*, *CiMA2* and *CiCA* (*Ciona intestinalis*), *BsMA2* and *BsCA1* (*Botryllus schlosseri*), *OlMA* and *OlCA* (*Oikopleura longicauda*), *BlMA* and *BlCA* (*Branchiostoma lanceolatum*), *PoMA* and *PoCA* (*Pisaster ochraceus*), *DmMA* and *DmCA* (*Drosophila melanogaster*) (for accession numbers see additional file 3). Amino acid position 1 (D) refers to the functional protein of human α-skeletal actin [85] (for this reference see additional file 4).
