# Supplementary material for: Muscle differentiation in a colonial ascidian: organisation, gene expression and evolutionary considerations
Source: BMC Dev Biol. 2009 Sep 8;9:48. doi: 10.1186/1471-213X-9-48 (PMC2753633; doi:10.1186/1471-213X-9-48)
Supplement: Additional file 7 — Figure S7. Alignment of troponin T sequences. [file 1471-213X-9-48-S7.pdf]

# Figure S7 (p 1/6)

```
            ....|....| ....|....| ....|....| ....|....| ....|....| ....|....| ....|....| ....|....| ....|....| ....|....|
                    10         20         30         40         50         60         70         80         90        100
AmTnT_hone  ---------- ---------- MSDDEEQYSS EEEVV----- ---------- ---------- ---------- ---------- ---------- ----------
BfTnT1a_Bu  ---------- ---------- MSDTEDV-EY EEQQ------ ---------- ---------- ---------- -EEEE----- ---------- --QEEEKEPT
BfTnT1b_Bu  ---------- ---------- MSDTEDV-EY EEQQ------ ---------- ---------- ---------- -EEEE----- ---------- --QEEE----
BflTnT_amp  ---------- ---------- ---------- ---------- ---------- ---------- ---------- ---------- ---------- ----------
BmTnT_Lepi  ---------- ---------- MSDEEEYSGS EEEEV----- ---------- ---------- ---------- ---------- ---------- ----------
BsTnT-c_Bo  ---------- ---------- MSDA------ ---------- ---------- ---------- ---------- -ENEEYEQQE QEEQEAPQ-- ----------
BtTnT1_Sk-  ---------- ---------- MSDAEEQ-EY EEEQ------ ---------- ---------- ---------- -PEEEE---- -AAEE----- -EEEAPEEPE
BtTnT2_Car  ---------- ---------- MSDVEEAVEE YEE--QEEAA EEE------- ---HEEAVEE EAG------- -GEAEAGEPC TAE------- DGEEEEGREA
BtTnT3_Sk-  ---------- ---------- MSD-EEVEHV E--------- ---------- ---------- ---------- -EEYEEEEEA QEE------- ----------
CiTnT_Cion  MSDSEEYSSE EETSEEESGE SSGEDGEEET GEIKRTS--- ---------- ---------- ---------- -QEEEIETQE VEEVEEEETT EEPE------
CjTnT3_Sk-  ---------- ---------- MSDTEEVEHG E--------- ---------- ---------- ---------- -EEYEEE--- -EEVQ----- -E--------
CnTnT_Biva  ---------- ---------- MDYDDE---- PRTGD----- ---------- ---------- ---------- ---------- ---------- ----------
Csp1TnT_po  ---------- ---------- MSDEEDQVIG SEEEG----- ---------- ---------- ---------- ---------- ---------- ----------
DmTnT_frui  ---------- ---------- MSDDEEYTSS EEEEV----- ---------- ---------- ---------- ---------- ---------- ----------
DpTnT_Daph  ---------- ---------- MSDEEEY--S DEEEV----- ---------- ---------- ---------- ---------- ---------- ----------
DrTnT1_Sk-  ---------- ---------- MCDTEEFVAE FEEEVKEQ-- ---------- ---------- --------EA EEEEEAEEEV KEEAEEKEEV EEEQEEAEEG
DrTnT2_Car  ---------- ---------- MSDNEE-VEE YEEQEEEQVE EEEE------ --VQEEAQHD E--------- --EAQQEENA GGDEE----- TTQEHDGEEE
DrTnT3a_Sk  ---------- ---------- MSDTEDIEQH FEEE------ ---------- ---------- ---------- ---------- ---------- ----------
DrTnT3b_Sk  ---------- ---------- MSDTEDVGVE EYDE------ ---------- ---------- ---------- ---------- ---------- ----------
FcTnT2_Car  ---------- ---------- MSDLEEVVEE YEEEQEEEAA EE-------- ---QEEAAAE EA-------- EGEAETEETN VEG------- DGQEEEAKET
GgTnT1_Sk-  ---------- ---------- MSEAEE--EY EEEQ------ ---------- ---------- ---------- -PEEEEEAAA AAEEE----- -EEEEAEASK
GgTnT2_Car  ---------- ---------- MSDSEEVVEE YEQEQEEEYV EEEEEEWLEE DDGQEDQVDE E--------- --EEETEETT AEEQEDETKA PGEGGEGDRE
GgTnT3_Sk-  ---------- ---------- MSDTEEVEHG E--------- ---------- ---------- ---------- -EEYEEEAHE AEEVH----- -E--------
GmTnT_Sk-f  ---------- ---------- MSDTEDVDQV EDEK------ ---------- ---------- ---------- ---------- ---------- ----------
HcTnT_Hyla  ---------- ---------- MSDTEEVEQV EEEYE----- ---------- ---------- ---------- -EEEAEEVEE HHEA------ ----------
HqTnT_Arac  ---------- ---------- --------MT EAEML----- ---------- ---------- ---------- ---------- ---------- ----------
HrTnT-a_Ha  ---------- ---------- MSGE------ ---------- ---------- ---------- ---------- -EEE--PQQH HEDVEKP--- ----------
HrTnT-l_Ha  ---------- ---------D STG------- ---------- ---------- ---------- ---------- ---------- ---------- ----------
HroTnT_Hir  ---------- ---------- MSDDES---- SHSEA----- ---------- ---------- ---------- ---------- ---------- ----------
HsTnT1_Sk-  ---------- ---------- MSDTEEQ-EY EEEQ------ ---------- ---------- ---------- -PEEE----- -AAEE----- -EEEAPEEPE
HsTnT2_Car  ---------- ---------- MSDIEEVVEE YEEEEQEEAA VEEEEDWRED EDEQEEAAEE DA-------- EAEAETEETR AEE------- DEEEEEAKEA
HsTnT3_Sk-  ---------- ---------- MSD-EEVEQV E--------- ---------- ---------- ---------- -EQYEEEEEA QEEA------ ------AEVH
MmTnT1_Sk-  ---------- ---------- MSDTEEQ-EY EEEQ------ ---------- ---------- ---------- -AEDE----- -EAVE----- -EEEAPEEPE
MmTnT2_Car  ---------- ---------- MSDAEEVVEE YEEEQEEEDW SEEEED---- --EQEEAVEE EEAGGAEPEP EGEAETEEAN VEE------- VGPDEEAKDA
MmTnT3_Sk-  ---------- ---------- MSD-EETEQV E--------- ---------- ---------- ---------- -EQYEEEEEA QEE------- -------EVQ
MtTnT3_Sk-  ---------- ---------- MSDTEEVEHG EAHEAEEVHE EAAKAH---- ---------- ---------- EEEAHEEAHA HEEAH----- -AH------H
OcTnT2_Car  ---------- ---------- MSDLEEVVEE YEEEQEAEEA AAEEEDWRED EDEQEAGEEE EAGGGR---- EAEAETEETQ AEE------- DGQEEEDKED
OcTnT3_Sk-  ---------- ---------- MSD-EEVEHV E--------- ---------- ---------- ---------- -EQYEEEEEA QEEAP----- -SPAEVHEPA
RnTnT1_Sk-  ---------- ---------- MSDTEEQ-EY EEEQ------ ---------- ---------- ---------- -AEDE----- -EAV------ -EEEAPEEPE
RnTnT2_Car  ---------- ---------- MSDAEEEVVE YEEEQEEEDW SEEEED---- --EQEEAVEE E-DGEAEPDP EGEAEAEEDK AEE------- VGPDEEARDA
RnTnT3_Sk-  ---------- ---------- MSD-EETEQV E--------- ---------- ---------- ---------- -EQYEEEEEA QEE------- -------EVQ
SaTnT-a_Sk  ---------- ---------- MSDTEEVDQV EEYD------ ---------- ---------- ---------- ---------- ---------- ----------
SaTnT-e_Sk  ---------- ---------- MSDTEEVDQV EEYDAVEEEV VEEVEV---- ---------- ---------- APEAAPEPEP EPEPE----- -PEPEPVVEP
SaTnT-l_Sk  ---------- ---------- MSDTEEVDQV EEEK------ ---------- ---------- ---------- ---------- ---------- ----------
SaTnT1_Sk-  ---------- ---------- MSDVEEEYEE QAEE------ ---------- ---------- ---------- -AEEEQEAEP EQEEE----- -AEQVEEDAG
SaTnT2_Sk-  ---------- ---------- MSD------- ---------- ---------- ---------- ---------- -TEEVLEEEV QDGED----- ----------
SaTnTi_Sk_  ---------- ---------- MSDSEEIVEE YEEEE----- ---------- ---------- --------EV EEEEEVEEEV EEEEEEQDNE TEKKEEQEE-
SpTnT1_sea  ---------- ---------- ---------- ---------- ---------- ---------- ---------- ---------- ---------- ----------
SpTnT2_sea  ---------- ---------- MNRYQLRIDT HARDCH---- ---------- ---------- ---------- -KRQEFKQEI IQALKMVRLV IEKYDEEIAA
SsTnT_Sk-f  ---------- ---------- MSDTEEVEAH R--------- ---------- ---------- ---------- ---------- ---------- ----------
StTnT1S_Sk  ---------- ---------- MADEE----- ---------- ---------- ---------- ---------- -NEEEVEVEV PAEEDAAPAE EEPEPEPEPD
XlTnT2a_Ca  ---------- ---------- MSDTEDIIEE YEEE------ ---------- -----DKVSE N--------- --EGGEEE-- ---------- PEEDGNEEEV
XlTnT2b_Ca  ---------- ---------- MSDTEDIIEE YEEE------ ---------- -----DKVSE N--------- --EGGEEE-- ---------- PEED------
XlTnT3_Sk-  ---------- ---------- MSDTEEVEQV EEYEE----- ---------- ---------- ---------- -EAQEEESQE QEEEP----- -VV-------
```

# Figure S7 (p 2/6)

```
             ....|....| ....|....| ....|....| ....|....| ....|....| ....|....| ....|....| ....|....| ....|....| ....|....|
                    110        120        130        140        150        160        170        180        190        200
AmTnT_hone   ---------- ------EETK QPEGRKI--- ---------- -EGRASQKES GENIEF-MK- RQEQKRSDLD E--------Q LKEYIAEWRK QRAKEEEELK
BfTnT1a_Bu   ---------- ----EEAGGE EERPRP---- ---------R PMMPRLAPPK IPEGE--RVD FDDIHRKRME KD-----LLE LQTLIDVHFG QRKKEEEELI
BfTnT1b_Bu   ---------- ---------E EERPRP---- ---------R PMMPRLAPPK IPEGE--RVD FDDIHRKRME KD-----LLE LQTLIDVHFG QRKKEEEELI
BflTnT_amp   ---------- ---------- ---------- ---------- ---------- ---------- ---MADKRRQ KD-----HVE LEEFIKKHFE KRAKEEEELQ
BmTnT_Lepi   ---------- ------EEEV PETKP----- ---------- --APKQ--EG EGDPEF-IK- RQDQKRSDLD E--------Q LKEYINEWRK QRAKEEDELK
BsTnT-c_Bo   ---------- ----EVAPEA VEKPRHS--- ---------- --MASITPLK LPEGE--KVE FDEISRKRHQ KD-----LEE LQALIAKHFD QRRAEEEELE
BtTnT1_Sk-   ---------- ----PAAEPE EERPKPS--- ---------R PVVPPLIPPK IPEGE--RVD FDDIHRKRME KD-----LLE LQTLIDVHFE QRKKEEEELV
BtTnT2_Car   ---------- ----EDGPVE EFKPKP---- --------RP FMP-NLVPPK IPDGE--RVD FDDIHRKRME KD-----LNE LQTLIEAHFE NRKKEEEELV
BtTnT3_Sk-   ---------- ---------- EEVQE----- --------EE KPRPRLTAPK IPEGE--KVD FDDIQKKRQN KD-----LME LQALIDSHFE ARKKEEEELV
CiTnT_Cion   ---------- ---EQTQQAT TPTPKLS--- ---------- --IPKLIPPK IPDGE--LVD LEDIHRKRME KD-----MVE LQGLINAHFE QRKKDEEEIE
CjTnT3_Sk-   ---------- ----EEVHEP APPPE----- ---------E KPRIKLTAPK IPEGE--KVD FDDIQKKRQN KD-----LIE LQALIDSHFE ARRKEEEELV
CnTnT_Biva   ---------- ------GNEA RLAMEEA--- ---------- -ARK------ ---------- ----KKEKVE S--------E IAEYEEMRRE QREKEAEDLE
Csp1TnT_po   ---------- ------GDEG QVEEEEE--- ---------- -SGDEQSAPA KDSGPS-EAQ LAMEKRRQHQ ASLGANLDEV AQAVLETSRI ERERLQEEIE
DmTnT_frui   ---------- ------VEET REETK----- ---------- --PPQTPAEG EGDPEF-IK- RQDQKRSDLD D--------Q LKEYITEWRK QRSKEEDELK
DpTnT_Daph   ---------- ------SKKP QKKGAP---- ---------- -APAHHEDEG PQGADV-LKH RQDQKRAELE I--------Q LKEYIDEWRK QRVKEEEDLK
DrTnT1_Sk-   -DEPEETHQE EADDEHEAEE DTKPKPK--- ---------- MFVPNIIPPK LPDGE--KVD FDDLHRKRVE KD-----FNE LQSLINLHFT TRQKEEDELV
DrTnT2_Car   ---------- ----TEDGGE EAKPKFL--- --------KP FMLPNLVPPK IPDGE--RVD FDDIHRKRME KD-----LNE LQTLIEAHFE SRKKEEEEII
DrTnT3a_Sk   ---------- ---------- ---------- ---------- KPKFKPTAPK IPDGE--KVD FDDIQKKRHN KD-----TLE LQCLIDAHFE HRQKEEEELI
DrTnT3b_Sk   ---------- ---------- ---------- --------EE KPKFKPSAPK IPDGD--KVD FDDIQKKRQN KD-----LVE LQALIDAHFE HRKKEEEELI
FcTnT2_Car   ---------- ----EDGPVE ESKPKP---- --------RP FMP-NLVPPK IPDGE--RVD FDDIHRKRME KD-----LNE LQTLIEAHFE NRKKEEEELI
GgTnT1_Sk-   ---------- ----PHEEPE EERPRP---- ---------R PVVPQLAPPK IPEGE--RVD FDDIHRKRME KD-----LLE LQTLIDAHFE QRRREENELV
GgTnT2_Car   ---------- ----QEPGEG ESKPKP---- --------KP FMP-NLVPPK IPDGE--RLD FDDIHRKRME KD-----LNE LQALIEAHFE SRKKEEEELI
GgTnT3_Sk-   ---------- ----EEVHEP APPPEEAPEE --------EE KPRIKLTAPK IPEGE--KVD FDDIQKKRQN KD-----LIE LQALIDSHFE ARRKEEEELV
GmTnT_Sk-f   ---------- ---------- ---------- ---------- -PKFKPSAPK IPDGE--KVD FDDIQKKRQN KD-----LSE LQGLIDAHFE GRKKEEEELI
HcTnT_Hyla   ---------- ---------- ---------- --------EE KPKPKITAPK IPDGE--KVD FDDIQKKRQN KD-----LIE LQSLIDQHFE GRKKEEEELI
HqTnT_Arac   ---------- ------MEEK ---------- ---------- ---------- -------LR- ---KKKEEEE E--------M WTEYLEQRKK QRAKEEDELR
HrTnT-a_Ha   ---------- ---------- --MPRHS--- ---------- --TTSIP--R LPDGE--KVD LDVITQKRHQ KD-----LEE LQALITAHFE QRKQEEEQLE
HrTnT-l_Ha   ---------- ---------- KVAPKFT--- ---------- --MPNITPPK IPDGE--VID LEDIHRKRMD KV-----LLE LQSLISSHFE TRKRAGGEID
HroTnT_Hir   ---------- ------ESEP EVPKKVE--- ---------- -PPKKRQEP- ---------- ----KRQQNS S----GLDES AKELLEFNRQ EREKMEDEID
HsTnT1_Sk-   ---------- ----PVAEPE EERPKPS--- ---------R PVVPPLIPPK IPEGE--RVD FDDIHRKRME KD-----LLE LQTLIDVHFE QRKKEEEELV
HsTnT2_Car   ---------- ----EDGPME ESKPKP---- --------RS FMP-NLVPPK IPDGE--RVD FDDIHRKRME KD-----LNE LQALIEAHFE NRKKEEEELV
HsTnT3_Sk-   ---------- ----EEVHEP EEVQEDT--- --AEEDAEEE KPRPKLTAPK IPEGE--KVD FDDIQKKRQN KD-----LME LQALIDSHFE ARKKEEEELV
MmTnT1_Sk-   ---------- ----PVAERE EERPKPS--- ---------R PVVPPLIPPK IPEGE--RVD FDDIHRKRME KD-----LLE LQTLIDVHFE QRKKEEEELI
MmTnT2_Car   ---------- ----EEGPVE DTKPKPS--- --------RL FMP-NLVPPK IPDGE--RVD FDDIHRKRVE KD-----LNE LQTLIEAHFE NRKKEEEELI
MmTnT3_Sk-   ---------- ----EEAPEP EEVQEDAVAE EEREEDEEEE KPRPKLTAPK IPEGE--KVD FDDIQKKRQN KD-----LME LQALIDSHFE ARKKEEEELI
MtTnT3_Sk-   ---------- ----EEVHEP APPPVHEP-- --------EE KPRIKLTAPK IPEGE--KVD FDDIQKKRQN KD-----LIE LQALIDSHFE ARRKEEEELV
OcTnT2_Car   ---------- ----EDGPVE ESKPKP---- --------RP FMP-NLVPPK IPDGE--RVD FDDIHRKRME KD-----LNE LQTLIEAHFE NRKKEEEELV
OcTnT3_Sk-   ---------- ----PEVHVP EEVHEDALED -MREEEEEEE KPRPKLTAPK IPEGE--KVD FDDIQKKRQN KD-----LME LQALIDSHFE ARKKEEEELV
RnTnT1_Sk-   ---------- ----PVAERE EERPKPS--- ---------R PVVPPLIPPK IPEGE--RVD FDDIHRKRME KD-----LLE LQTLIDVHFE QRKKEEEELI
RnTnT2_Car   ---------- ----EDGPVE DSKPKPS--- --------RL FMP-NLVPPK IPDGE--RVD FDDIHRKRME KD-----LNE LQTLIEAHFE NRKKEEEELI
RnTnT3_Sk-   ---------- ----EEAPEP EEVQE----- --------EE KPRPKLTAPK IPEGE--KVD FDDIQKKRQN KD-----LME LQALIDSHFE ARKKEEEELI
SaTnT-a_Sk   ---------- ---------- ---------- --------EE KPKFKPSAPK IPDGE--KVD FDDIQKKRQN KD-----LVE LQALIDAHFE CRKKEEEELI
SaTnT-e_Sk   ---------- ----EPEPEP EPEPEPEEAV -----EEEEE KPKFKPSAPK IPDGE--KVD FDDIQKKRQN KD-----LVE LQALIDAHFE CRKKEEEELI
SaTnT-l_Sk   ---------- ---------- ---------- ---------- -PKFKPSAPK IPDGE--KVD FDDIQKKRQN KD-----LVE LQALIDAHFE CRKKEEEELI
SaTnT1_Sk-   ---------- ----EQQEYQ EERPKP---- ---------K PMVPQLAPPK IPEGD--RVD FDDIHRKRME KD-----LLE LHTLIDVHFE QRKRDEEELI
SaTnT2_Sk-   ---------- ---------- ESKPKPK--- ---------- -FMTNISAPK IPDGE--KVD FDDIHRKRQE KD-----LSE LQSLIEAHFI QRKKEEEELI
SaTnTi_Sk_   ---------- ---------- ESKPRHKT-- ---------- TYVPNIAPPK LPDGE--KVD FDDLHRKRVE KD-----FND LQSLIEVHFS SRQKEEEELI
SpTnT1_sea   ---------- ---------- ---------- -------MAE TDPETMTDED RQQAE----- -----EKKKE FE------AE VEEARSDLKK VIDQYDEEIN
SpTnT2_sea   LKKHRLERKN EQAMWLDRIK KNEEDRKKRR QEENERLIRM REQKKIEREE RQRAMRNPLA YKNTVQKRQE FK------QE IIQALKMVRL VIEKYDEEIA
SsTnT_Sk-f   ---------- ---------- ---------- ---------- -PQFK--APK IPDGE--KVD FDDIQKKRQN KD-----LVE LQGLIDAHFE HRKKEEEELI
StTnT1S_Sk   IEEPTAVEET PAEEASGETQ DSKAKPK--- ---------- SFMPNVAPPK LPEGDG-KVD FDDLHRKRQE KD-----MAE LTSLIESHFV QRKKDEDELI
XlTnT2a_Ca   ---------- ----NEEEEE ESKPKP---- --------KL FMP-NLMPLK IPDGE--KVD FDDIHRKRME KD-----LTE LQTLIEAHFE SRKKEEEELE
XlTnT2b_Ca   ---------- ---------E ESKPKP---- --------KL FMP-NLMPLK IPDGE--KVD FDDIHRKRME KD-----LTE LQTLIEAHFE SRKKEEEELE
XlTnT3_Sk-   ---------- ----EEAPQV EEAYEEAEEE -EHGEEYDEE KPKPKLTAPK IPDGE--KVD FDDIQKKRQN KD-----LIE LQSLIDTHFE ARKKEEEDII
```

# Figure S7 (p 3/6)

```
             ....|....| ....|....| ....|....| ....|....| ....|....| ....|....| ....|....| ....|....| ....|....| ....|....|
                   210        220        230        240        250        260        270        280        290        300
AmTnT_hone   RLKEKQAKRK ITRADEEKRL AQKKKEEEER RQREIEEKKQ RDMEEKRKRL EESEKKRQAM MQAMKEQ-AS KKGPNFTITR K--------- DLAGN----L
BfTnT1a_Bu   GLKDRIENRR SERAEQQR-- -FRTEKERER QARMAEEKMR KEEEEAKKRA DDDAKKKKVL SNMGAQYGGY LTK---AEQK RGK------- ----------
BfTnT1b_Bu   GLKDRIENRR SERAEQQR-- -FRTEKERER QARMAEEKMR KEEEEAKKRA DDDAKKKKVL SNMGAQYGGY LTK---AEQK RGK------- ----------
BflTnT_amp   LLIVRREKRK AEMADKET-- -ARKERERER VNEEKARLMK QREEEERKRK EEDDRKK-KL ANVNLHIGGY LSRLEGKEKG PSK------- ----------
BmTnT_Lepi   RLKEKQAKRK VSRAEEEKRL AQKKKEEEER RVREIEEKKQ RDIEEKRQRL EEAEKKRQAM LQAMKD--AS KTGPNFTIQK K--------- SENFG----L
BsTnT-c_Bo   VLRLKLEKRK EERAERNR-- -LRQQKEKER LAREREERKL KEQEEEKKRQ EEEEKKRKAI ANMSLHYGGY LAR---AEKN KPN------- ----------
BtTnT1_Sk-   ALKERIERRR AERAEQQR-- -FRTEKERER QAKLAEEKMR KEEEEAKKRA EDDAKKKKVL SNMGAHFGGY LVK---AEQK RGK------- ----------
BtTnT2_Car   SLKDRIEKRR AERAEQQR-- -IRAEREKER QTRLAEERAR REEEESRRKA EDEARKKKAL SNMMHFGG-Y IQK-AQTERK SGK------- ----------
BtTnT3_Sk-   ALKERIEKRR AERAEQQR-- -IRAEKERER QNRLAEEKAR REEEDAKRRA EDDLKKKKAL SSMGANYSSY LAK---ADQK RGK------- ----------
CiTnT_Cion   ELRVRIEERK SKRAEQIR-- -IRQDRERER MAQEREERKR KEEEEESKRQ EEEARKKAAI ANMSLHYGGY LAR---AEKN KPN------- ----------
CjTnT3_Sk-   ALKERIEKRR AERAEQQR-- -IRAEKEKER QARLAEEKAR REEEDAKRKA EDDLKKKKAL SSMGASYSSY LAK---ADQK RGK------- ----------
CnTnT_Biva   QLRLKREQRK QERIEEDRRL LEIRKEEDKR RKAEEEERKR KKQEDERKRI EA----KKAK LKELEERKKM SKTPNFVITK K--------- GASNLEEASK
Csp1TnT_po   EMKSRNEERK RIREEEEKAL AEKRAEEDAQ RKAAEEERGR KKQEEEMKRR EM----RAAR LAAYEALS-L PQGRNFVITK K--------D GGEEQEEELD
DmTnT_frui   KLKEKQAKRK VTRAEEEQKM AQRKKEEEER RVREAEEKKQ REIEEKRMRL EEAEKKRQAM LQAMKD--KD KKGPNFTIAK K--------- DAGLG----L
DpTnT_Daph   RLKEKQAKRK VLREEEDKKL ATMKKEEEDR LRRLVEEKKQ GDLEEKRRRL AEVEQKRQVA DKVSKD--KA AISSNFKVVK KGTVDPTA-A QASTGGMDKF
DrTnT1_Sk-   ALKNRIERRR TDRADQQR-- -IRTERDRER QARLAEERAR REEEAAKLRA EEDARKKKIL S--NKGYGGF LQK---VDQK KG-------- ----------
DrTnT2_Car   SLKDRIEKRR SERAEQQR-- -IRSERERER QKRLEEERAR KEEEEAKKRA EDDAKKKKTL T-SLHFGG-Y MQK---IERR SGK------- ----------
DrTnT3a_Sk   ALRERIEKRR SERAEQQR-- -IRTEQEKER HARREEERLR KEEADAKKKA EEDAKKKSAL SSMGSNYSSY LQK---ADSK KGG------- ----------
DrTnT3b_Sk   ALKDRIEKRR SERAEQQR-- -IRAEKDKER QARREEERLR KEEADAKKRA DEDAKKKSAL SNMGSQYSSY LQK---ADSK RGG------- ----------
FcTnT2_Car   SLKDRIEKRR AERAEQQR-- -IRNEREKER QTRLAEERAR REEEENRRKA EDEARKKKAL SNMMHFGG-Y IQK---TERK SGK------- ----------
GgTnT1_Sk-   ALMERIERRR AERNEQLR-- -SRTEKERER QARLAEEKLR KEEEEAKKRA EDDAKKKKVL SNM-PHFGGY LAK---AEQR RGK------- ----------
GgTnT2_Car   SLKDRIEQRR AERAEQQR-- -IRSEREKER QARMAEERAR KEEEEARKKA EKEARKKKAF SNMLHFGG-Y MQK---SEKK GGK------- ----------
GgTnT3_Sk-   ALKERIEKRR AERAEQQR-- -IRAEKEKER QARLAEEKAR REEEDAKRKA EDDLKKKKAL SSMGASYSSY LAK---ADQK RGK------- ----------
GmTnT_Sk-f   ALKERIEKRR AERAEQQR-- -IRSEKDKER QARREEERLK REEADAKKKM EEDAKKKSAL SNMGSNYSSH LQK---ADQK RGGG------ ----------
HcTnT_Hyla   GLKERIEKRR AERSEQQR-- -IRAEKEKER QNRLAEEKAR REEQDAMRRA EDDMKKKKAL SSMGANYSSY LAK---ADQK RGK------- ----------
HqTnT_Arac   KLKERQAKRK AQRAEQEAKL MEFKKKQEEQ RIREMEEKKA REAEAKRKRL EEAERKRQAM QAAKEKREQE PVKPNFVITK REGEEMGGGS ALGSSGFDKF
HrTnT-a_Ha   ELRLRLEKRK EERAERLR-- -IREEKTKER VAREREERKH KEEEDEKKRK EEEEKKKAAI ANMSLHYGGY LAR---AEKN KPN------- ----------
HrTnT-l_Ha   QLRIRIEERK ACREEQMR-- -VRQEKEKER MAREREDRKR KEEEEGQKRF EEEAKRKAAI ANMSLHYGGY LAR---PIKI SPN------- ----------
HroTnT_Hir   ELRRRSEKRK KQREIEEKRL TAERAAEDER RKAAEEAKRR QKIEDEEKKQ KD----RARK MAEFEKWK-Q PQKRNFVITR KA-------D GDEEDEEDKE
HsTnT1_Sk-   ALKERIERRR SERAEQQR-- -FRTEKERER QAKLAEEKMR KEEEEAKKRA EDDAKKKKVL SNMGAHFGGY LVK---AEQK RGK------- ----------
HsTnT2_Car   SLKDRIERRR AERAEQQR-- -IRNEREKER QNRLAEERAR REEEENRRKA EDEARKKKAL SNMMHFGG-Y IQKQAQTERK SGK------- ----------
HsTnT3_Sk-   ALKERIEKRR AERAEQQR-- -IRAEKERER QNRLAEEKAR REEEDAKRRA EDDLKKKKAL SSMGANYSSY LAK---ADQK RGK------- ----------
MmTnT1_Sk-   ALKDRIERRR AERAEQQR-- -FRTEKERER QAKLAEEKMR KEEEEAKKRA EDDAKKKKVL SNMGAHFGGY LVK---AEQK RGK------- ----------
MmTnT2_Car   SLKDRIEKRR AERAEQQR-- -IRNEREKER QNRLAEERAR REEEENRRKA EDEARKKKAL SNMMHFGG-Y IQKQAQTERK SGK------- ----------
MmTnT3_Sk-   ALKERIEKRR AERAEQQR-- -IRAEKEREP QNRLAEEKAR REEEDAKRRA EDDMKKKKAL SSMGANYSSY LAK---ADQK RGK------- ----------
MtTnT3_Sk-   ALKERIEKRR AERAEQQR-- -IRAEKEKER QARLAEEKAR REEEDAKRKA EDDLKKKKAL SSMGATYSSY LAK---ADQK RGK------- ----------
OcTnT2_Car   SLKDRIEKRR AD-AEQLR-- -IRAEREKER QNRLAEERAR REEEESRRKA EDEARKKKAL SNMMHFGG-Y IQKQAQTERK SGK------- ----------
OcTnT3_Sk-   ALKERIEKRR AERAEQQR-- -IRAEKERER QNRLAEEKAR REEEDAKRRA EEDLKKKKAL SSMGANYSSY LAK---ADQK RGK------- ----------
RnTnT1_Sk-   ALKDRIERRR AERAEQQR-- -FRTEKERER QAKLAEEKMR KEEEEAKKRA EDDAKKKKVL SNMGAHFGGY LVK---AEQK RGK------- ----------
RnTnT2_Car   SLKDRIEKRR AERAEQQR-- -IRNEREKER QNRLAEERAR REEEENRRKA EDEARKKKAL SNMMHFGG-Y IQK-AQTERK SGK------- ----------
RnTnT3_Sk-   ALKERIEKRR AERAEQQR-- -IRAEKERER QNRLAEEKAR REEEDAKRRA EDDLKKKKAL SSMGANYSSY LAK---ADQK RGK------- ----------
SaTnT-a_Sk   ALKDRIEKRR AERAEQQR-- -VRAEKEKER QARREEERRI REEADAKKKA DEDAKKKSAL SSMGSNYSSH LQR---ADQK RGG------- ----------
SaTnT-e_Sk   ALKDRIEKRR AERAEQQR-- -VRAEKEKER QARREEERRI REEADAKKKA DEDAKKKSAL SSMGSNYSSH LQR---ADQK RGG------- ----------
SaTnT-l_Sk   ALKDRIEKRR AERAEQQR-- -VRAEKEKER QARREEERRI REEADAKKKA DEDAKKKSAL SSMGSNYSSH LQR---ADQK RGG------- ----------
SaTnT1_Sk-   SLKDRIERRR SERAEIQR-- -VRAEKEKDR QNRIAEERHR KEEEEAKKKA DDDAKKKKVL SGMGANFGGF LAK---AESR RGK------- ----------
SaTnT2_Sk-   ALVNRIEKRR AERAEQQR-- -VRTEREKER QARLAEEKER KEQEEQRKKY DDDAKKKKAL SNMTQQYS-A GQK---SESR KGG------- ----------
SaTnTi_Sk_   ALRSRIERRR ADRAEQQR-- -VRSEQDRER QTRQAEERAR REEEAAKLRA EEEAKKKAIF T--NKSFGGY LQK---VDQK KG-------- ----------
SpTnT1_sea   VIREKQASRR AEQAMWLD-- -RIKKNEEAR RLRHEEENRR IKEAQEKKKK EREERKRAMQ NPNKFQPIIQ DERVRFAKMT ---------- ----------
SpTnT2_sea   ALKKHRLERK NEQAMWLD-- -RIKKNEEDR KKRRQEENER LIRMREQKKI EREERQRAMR NPLAYKNTVQ DERIRFARMT ---------- ----------
SsTnT_Sk-f   SLKERIEKRR AERAEQNR-- -IRSEKEKER AARREEERLK REEADAKKKA DEDAKKKSAL SSMGSNYSSH LQK---ADSK RGG------- ----------
StTnT1S_Sk   SLVNRIEKRR TERAEQQR-- -IRAEQEKER QARLAEEKER REQDEQRRKH DDDAKKKKAL TTMTHTYGGI QQK---QEGK KGA------- ----------
XlTnT2a_Ca   ALTERMEKRR AERAEQLR-- -IRTEREKER QARVAEERAR KEEEENRKRA QDDDRKKKAF SNMLHFGG-Y LQK---TERK VGK------- ----------
XlTnT2b_Ca   ALTERMEKRR AERAEQLR-- -IRTEREKER QARVAEERAR KEEEENRKRA QDDDRKKKAF SNMLHFGG-Y LQK---TERK VGK------- ----------
XlTnT3_Sk-   ALKDRIEKRR AERAEQLR-- -IRTEKEKER QTRLAEEKAR REEQDLMRRA EDDMKKKKAL SSMGATYSSY LAK---ADQK RGK------- ----------
```

# Figure S7 (p 4/6)

```
            ....|....| ....|....| ....|....| ....|....| ....|....| ....|....| ....|....| ....|....| ....|....| ....|....|
                   310        320        330        340        350        360        370        380        390        400
AmTnT_hone  TSAQLERN-- -KTKEQLEEE KKISLSIRIK PLEIDGFSIE -KLR------ ---------- SKANELWDTI VKLETEKYDL EERQKRQDYD LKELKERQKQ
BfTnT1a_Bu  ---------- ---RQTGREM KRKILAERHK PLVIDNMNDN -QLR------ ---------- ERAKELTDWM YQLESEKFDL MEKMKTQKYE INVLYNRI-S
BfTnT1b_Bu  ---------- ---RQTGREM KRKILAERHK PLVIDNMNDN -QLR------ ---------- ERAKELTDWM YQLESEKFDL MEKMKTQKYE INVLYNRI-S
BflTnT_amp  ---------- --RQLAQEAK RKKIMARVKA LPDLGTLSDH -ELR------ ---------- EKAQELQDRL KFSEEELYDM KDKVQRQVYD VSTTRIRCLQ
BmTnT_Lepi  SNAQLERN-- -KTKEQLEEE KKISLSIRIK PLTIEGLSVD -KLR------ ---------- QKAQELWECI VKLETEKYDL EERQKRQDYD LKELKERQKQ
BsTnT-c_Bo  ---------- --KRQTEREK KKKILADRKK PLNVDHMNIN -QLR------ ---------- EKAKELWDYL YTLEEEKIDA ETRIDRQKYD LNQLRQRVNE
BtTnT1_Sk-  ---------- ---RQTGREM KLRILSERKK PLNIDHMGEE -QLR------ ---------- EKAQELSDWI HQLESEKFDL MAKLKQQKYE INVLYNRI-S
BtTnT2_Car  ---------- ---RQTEREK KKKILAERRK VLAIDHLNED -QLR------ ---------- EKAKELWQMI YDLEAEKFDL QEKFKQQKYE INVLRNRI-N
BtTnT3_Sk-  ---------- ---KQTAREM KKKVLAERRK PLNIDHLSED -KLR------ ---------- DKAKELWDTL YQLETDKFEY GEKLKRQKYD ITNLRSRI-D
CiTnT_Cion  ---------- --KRQTDREK KKKLLADRRK PLNIDHLSDD -KLK------ ---------- EKAQELWDNL YKLEEEKYDH EQRINRQKYD INQLRQRVNE
CjTnT3_Sk-  ---------- ---KQTARET KKKVLAERRK PLNIDHLNED -KLR------ ---------- DKAKELWDWL YQLQTEKYDF TEQIKRKKYE ILTLRCRL-Q
CnTnT_Biva  DMA------- -KSKEQLEEE KRAILAQRIQ PLTVDGLDLA -ALM------ ---------- EKATEFHNKI KSLANEKYEL EERFKSQQYD MIELAERARQ
Csp1TnT_po  EEELANKEK- -KSKEQQEQE KKAILSQRIK PVNMDELNSS DKLK------ ---------- EKARELHNQI VRLESEKYDL EKRFKAQQYD MMELAERARQ
DmTnT_frui  SSAAMERN-- -KTKEQLEEE KKISLSFRIK PLAIEGFGEA -KLR------ ---------- EKAQELWELI VKLETEKYDL EERQKRQDYD LKELKERQKQ
DpTnT_Daph  SNVESARNDL LKSKEQLEEE KKISLSFRIK PLEIDGLSCD -GLR------ ---------- KKAAELWQTI VTLETEKYDL EERSKRQDYD LKELKERQRQ
DrTnT1_Sk-  ---------- --KKLTEREK KTKCLLERRK PLNIDHLNQE -KLG------ ---------- EKALDLWKWL NQLHAEKFEL GEKLKSQKYE IKVLRNRV-S
DrTnT2_Car  ---------- ---KQTEREK KKKIFGDRRK PLDIDNANES -ALR------ ---------- EKAKELWSWM RELEAEKFEL QYQFGKQKYE INVLRNRV-S
DrTnT3a_Sk  ---------- --KKQTEREK KKKILAERRK QLNIDHLNED -KLR------ ---------- DKAQELYEWI KTLESEKFEH MERLKRQKYE VTTLRRRV-E
DrTnT3b_Sk  ---------- --KKQTEREK KKKILAERRK PLNIDHMNED -KLR------ ---------- EKAKELWDWL YSLEAEKFEH MEKLKRQKYE VTTQRKRV-E
FcTnT2_Car  ---------- ---RQTEREK KKKILAERRK VLAIDHLNED -QLR------ ---------- EKAKELWQSI YNLEAEKFDL QEKFKQQKYE INVLRNRI-N
GgTnT1_Sk-  ---------- ---RQTGREM KLRILAERKK PLHIEHMRED -ELR------ ---------- AKAKELHDWI QQLESEKFDL MEKLRRQKYE INVLYNRI-S
GgTnT2_Car  ---------- ---KQTEREK KKKILSERRK PLNIDHLSED -KLR------ ---------- DKAKELWQTI RDLEAEKFDL QEKFKRQKYE INVLRNRV-S
GgTnT3_Sk-  ---------- ---KQTARET KKKVLAERRK PLNIDHLNED -KLR------ ---------- DKAKELWDWL YQLQTEKYDF AEQIKRKKYE IVTLRNRI-D
GmTnT_Sk-f  ---------- --KKETEREK KKKILAARRK GLNIDHLNED -KLK------ ---------- DKINELHEWM STLESEKFDH MERLNRQKYE VTTLRKRV-E
HcTnT_Hyla  ---------- ---KQTAREQ KKKILADRRK PLNVDHMSED -KLR------ ---------- EKAKELWDWL YQLESEKFEF GEKLKKQKYE VTTLRNRI-D
HqTnT_Arac  ANIMYARGEM GKTKEQMEED KKAILSFRIK PLEIEGLSVE -QLR------ ---------- EKAKQLWETV VSLESEKYDL EERQKRQDYD LKELAERQRQ
HrTnT-a_Ha  ---------- --KRQTEREK KKKLLAERKK NISVDHLSPD -KLR------ ---------- EKAQELWDLL YSLEEEKIDY EVRIDRQKYD LNQLRQRVNE
HrTnT-l_Ha  ---------- --KRQTDREK KKKILSDRRK PLNVDHLSAE -KLQ------ ---------- EKASELWKWL YSLEEEKYDF ETRIERQKYD INQLRQRVNE
HroTnT_Hir  NEEKSDAEKS KKTKEQLEAE KKAILRQRIK PLDIDGLDQA -KLA------ ---------- EKAKEFHSWL ARLEGERYDL EKRFKSQQAA LVDLAERARQ
HsTnT1_Sk-  ---------- ---RQTGREM KVRILSERKK PLDIDYMGEE -QLRARSAWL PPSQPSCPAR EKAQELSDWI HQLESEKFDL MAKLKQQKYE INVLYNRI-S
HsTnT2_Car  ---------- ---RQTEREK KKKILAERRK VLAIDHLNED -QLR------ ---------- EKAKELWQSI YNLEAEKFDL QEKFKQQKYE INVLRNRI-N
HsTnT3_Sk-  ---------- ---KQTAREM KKKILAERRK PLNIDHLGED -KLR------ ---------- DKAKELWETL HQLEIDKFEF GEKLKRQKYD ITTLRSRI-D
MmTnT1_Sk-  ---------- ---RQTGREM KLRILSERKK PLNIDYMGED -QLR------ ---------- EKAQELSEWI HQLESEKFDL MEKLKQQKYE INVLYNRI-S
MmTnT2_Car  ---------- ---RQTEREK KKKILAERRK ALAIDHLNED -QLR------ ---------- EKAKELWQSI HNLEAEKFDL QEKFKQQKYE INVLRNRI-N
MmTnT3_Sk-  ---------- ---KQTAREM KKKILAERRK PLNIDHLSDD -KLR------ ---------- DKAKELWDTL YQLETDKFEF GEKLKRQKYD ITTLRSRI-D
MtTnT3_Sk-  ---------- ---KQTARET KKKILAERRK PLNIDHLNED -KLR------ ---------- DKAKELWDWL YQLETEKYDF AEQIKRKKYE IVTLRNRI-D
OcTnT2_Car  ---------- ---RQTEREK KKKILAERRK VLAIDHLNED -QLR------ ---------- EKAKELWQSI YNLEAEKFDL QEKFKQQKYE INVLRNRI-N
OcTnT3_Sk-  ---------- ---KQTAREM KKKILAERRK PLNIDHLSDE -KLR------ ---------- DKAKELWDTL YQLETDKFEF GEKLKRQKYD IMNVRARV-E
RnTnT1_Sk-  ---------- ---RQTGREM KLRILSERKK PLNIDYMGED -QLR------ ---------- EKAQELSEWI HQLESEKFDL MEKLKQQKYE INVLYNRI-S
RnTnT2_Car  ---------- ---RQTEREK KKKILAERRK VLAIDHLNED -QLR------ ---------- EKAKELWQSI HNLEAEKFDL QEKFKQQKYE INVLRNRI-N
RnTnT3_Sk-  ---------- ---KQTAREM KKKILAERRK PLNIDHLSDD -KLR------ ---------- DKAKELWDTL YQLETDKFEF GEKLKRQKYD ITTLRSRI-D
SaTnT-a_Sk  ---------- --KKETEREK KKKILAARRK QLNIDHLNED -KLK------ ---------- DKINELHEWM VTLESEKFDH MERLKRQKYE VTTLRKRI-E
SaTnT-e_Sk  ---------- --KKETEREK KKKILAARRK QLNIDHLNED -KLK------ ---------- DKINELHEWM VTLESEKFDH MERLKRQKYE VTTLRKRI-E
SaTnT-l_Sk  ---------- --KKETEREK KKKILAARRK QLNIDHLNED -KLK------ ---------- DKINELHEWM VTLESEKFDH MERLKRQKYE VTTLRKRI-E
SaTnT1_Sk-  ---------- ---RLTGKEI RKKTLAERRQ PLGIDNLRED -GLK------ ---------- QRAQEMWNSI YQLESEKFDF IEHMKHQRYE IIVLLNRI-Q
SaTnT2_Sk-  ---------- --KKQTEREK KKKILADRRK ALNVDHLNED -KLK------ ---------- EKASELWQWL MGLEAEKFDL SEKLKRQKYD INQLLARV-Q
SaTnTi_Sk_  ---------- --KKLTAREE KKKALMDRKK PLNIDHLNQE -KLA------ ---------- EKAQDLWQWL HQLHAEKFEL AEKLKRQKYD IYVLRNRV-S
SpTnT1_sea  ---------- ---PAELRKE KEETLAKRVP VLDPESMSSD EGMK------ ---------- TLATEIHTRI MKAFGALFDL QEKEKRQKYD IKELTTRIES
SpTnT2_sea  ---------- ---VEELAKE KEETLAKRAP ALDLDSLGSE EAMK------ ---------- EAARDLYAKI VKAFGNLFDL QQTEKRQKYD IKELNTRINA
SsTnT_Sk-f  ---------- --KKETEREK KKKILASRRK VLNIDHLNEE -KLK------ ---------- EKAKELHEWM QTLESEKFDN MERLKRQKYE VTTLRKRV-E
StTnT1S_Sk  ---------- --KKQTEREK KKKILADRRK ALIIDHLNED -KLK------ ---------- EKANELWQWM MELEARSSTL SEKLKKQKYD INQLLARV-Q
XlTnT2a_Ca  ---------- ---KQTEREK KKMILAERKK PLNVENLNED -KLR------ ---------- TEAQHLFNRI YQLEAEKFDH QDTFKKQKYE INVLRNRV-S
XlTnT2b_Ca  ---------- ---KQTEREK KKMILAERKK PLNVENLNED -KLR------ ---------- TEAQHLFNRI YQLEAEKFDH QDTFKKQKYE INVLRNRV-S
XlTnT3_Sk-  ---------- ---KQTAREQ KKKILADRRK PLNVDHMNDD -KLR------ ---------- EKAKEMWDWL YQLEFEKFEM GEKLKKQKFE ITTLHRRV-E
```

# Figure S7 (p 5/6)

```
              ....|....| ....|....| ....|....| ....|....| ....|....| ....|....| ....|....| ....|....| ....|....| ....|....|
                     410        420        430        440        450        460        470        480        490        500
AmTnT_hone    QLRH-----K ALKKGLDPE- ---------- --ALTGKYPP KIQVASKYER RVDTRSYDDK KKLFEGGLTE QQKEFIEKQW AQQKEQFLGR QKTKLPKWFG
BfTnT1a_Bu    HAQKFK---- ---------- ---------- ---KGAG--- KGRVGGRWK- ---------- ---------- ---------- ---------- ----------
BfTnT1b_Bu    HAQKFK---- ---------- ---------- ---KGAG--- KGRVGGRWK- ---------- ---------- ---------- ---------- ----------
BflTnT_amp    S--------- ---------- ---------- ---------- ---------- ---------- ---------- ---------- ---------- ----------
BmTnT_Lepi    QLRH-----K ALKKGLDPE- ---------- --ALTGKHPP KIQVASKYER RVDTRSYDDK KKLFEGDLEK LNKDFLEKVW QERAEQFGGR QKARLPKWFG
BsTnT-c_Bo    YMGKYSKN-- ---------- -----K---- AKVKVAGHGG VAKTASAFK- ---------- ---------- ---------- ---------- ----------
BtTnT1_Sk-    HAQKFR---- ---------- ---------- ---KGAG--- KGRVGGRWK- ---------- ---------- ---------- ---------- ----------
BtTnT2_Car    DNQKVS---- ---------- ---------- ------KTRG KAKVTGRWK- ---------- ---------- ---------- ---------- ----------
BtTnT3_Sk-    QAQKHS---- ---------- ---------- ---KKAGTAP KGKVGGRWK- ---------- ---------- ---------- ---------- ----------
CiTnT_Cion    YMGKFSKS-- ---------- -----KRNQP GKKIAGHQG- IGAAASNFK- ---------- ---------- ---------- ---------- ----------
CjTnT3_Sk-    ELSKFS---- ---------- ---------- ---KKAG--A KGKVGGRWK- ---------- ---------- ---------- ---------- ----------
CnTnT_Biva    MNKG-----K KRAVQVDDS- -------YDP MAEKYGSCPP KVQMYSKYER HTDLRTYGTR VDYFETKAKK IEAEMAIGR- --KKEEDNLL KTMEETEETS
Csp1TnT_po    MNKVGGKKPK RLWHGHGESQ TPSVGAQVDK IQERYAGAPA KIEMYSRYER QKDKRTFSDR HVVFHGPTWK YPAKRIRACK IVKWDEDSGL PIYVEMEGAD
DmTnT_frui    QLRH-----K ALKKGLDPE- ---------- --ALTGKYPP KIQVASKYER RVDTRSYDDK KKLFEGGWDE ISKDSNEKIW NEKKEQYTGR QKSKLPKWFG
DpTnT_Daph    QNRQ-----K ALKLGLDPE- ---------- --ALTGKYPP KIALASKFER RVDHRTYSDK KDLFAGGWDV IVKDVDEKSW QEKYTEYTTR PKTKLPKWFG
DrTnT1_Sk-    DHQ--KGT-- ---------- -----K---- ---VAKTTR- -----KSWK- ---------- ---------- ---------- ---------- ----------
DrTnT2_Car    DHQKTS---- ---------- ---------- ---------- KRTKRGLRK- ---------- ---------- ---------- ---------- ----------
DrTnT3a_Sk    ELSKFS---- ---------- ---------- ---KKGAAAR RRK------- ---------- ---------- ---------- ---------- ----------
DrTnT3b_Sk    ELSKYS---- ---------- ---------- ---KKGAAAR RRK------- ---------- ---------- ---------- ---------- ----------
FcTnT2_Car    DNQKVS---- ---------- ---------- ------KTRG KAKVTGRWK- ---------- ---------- ---------- ---------- ----------
GgTnT1_Sk-    HAQKFK---- ---------- ---------- ---KVVG--- KGRVGGRWK- ---------- ---------- ---------- ---------- ----------
GgTnT2_Car    DHQKVK---- ---------- -----G---- ---SKAAR-G KTMVGGRWK- ---------- ---------- ---------- ---------- ----------
GgTnT3_Sk-    QAQKHS---- ---------- ---------- ---KKAG--A KGKVGGRWK- ---------- ---------- ---------- ---------- ----------
GmTnT_Sk-f    ELSKFS---- ---------- ---------- ---KKGKTVR RK-------- ---------- ---------- ---------- ---------- ----------
HcTnT_Hyla    QLQKHS---- ---------- ---------- ---KKASG-G KGKVGGRWK- ---------- ---------- ---------- ---------- ----------
HqTnT_Arac    INRS-----R ALKKGLDPE- ---------- --ALQGKHPP KIQVASKYER RTDRRTFTDK RELFAGGLEA MTDAEMEKMW EEKMMTFKEN ARQGLPKWDP
HrTnT-a_Ha    YMGKYSKN-- ---------- -----K---- SKVKVAGHGG VMKAASAFK- ---------- ---------- ---------- ---------- ----------
HrTnT-l_Ha    YMGKFSKT-- ---------- -----KRQTP GKVNTGARGG VSSKTGVFS- ---------- ---------- ---------- ---------- ----------
HroTnT_Hir    ANKVGKGGLK RIG-GADDS- -------VDK IQAKYAGAPA KVEMFSKYER QKDKRSYDDK YQIFTGPQFI SPVDRIKPQK ILHWDEER-M PIY----GAG
HsTnT1_Sk-    HAQKFR---- ---------- ---------- ---KGAG--- KGRVGGRWK- ---------- ---------- ---------- ---------- ----------
HsTnT2_Car    DNQKVS---- ---------- ---------- ------KTRG KAKVTGRWK- ---------- ---------- ---------- ---------- ----------
HsTnT3_Sk-    QAQKHS---- ---------- ---------- ---KKAGTPA KGKVGGRWK- ---------- ---------- ---------- ---------- ----------
MmTnT1_Sk-    HAQKFR---- ---------- ---------- ---KGAG--- KGRVGGRWK- ---------- ---------- ---------- ---------- ----------
MmTnT2_Car    DNQKVS---- ---------- ---------- ------KTRG KAKVTGRWK- ---------- ---------- ---------- ---------- ----------
MmTnT3_Sk-    QAQKHS---- ---------- ---------- ---KKAGATA KGKVGGRWK- ---------- ---------- ---------- ---------- ----------
MtTnT3_Sk-    QAQKHS---- ---------- ---------- ---KKAG--T KGKVGGRWK- ---------- ---------- ---------- ---------- ----------
OcTnT2_Car    DNQKVS---- ---------- ---------- ------KTRG KAKVTGRWK- ---------- ---------- ---------- ---------- ----------
OcTnT3_Sk-    MLAKFS---- ---------- ---------- ---KKAGTTA KGKVGGRWK- ---------- ---------- ---------- ---------- ----------
RnTnT1_Sk-    HAQKFR---- ---------- ---------- ---KGAG--- KGRVGGRWK- ---------- ---------- ---------- ---------- ----------
RnTnT2_Car    DNQKVS---- ---------- ---------- ------KTRG KAKVTGRWK- ---------- ---------- ---------- ---------- ----------
RnTnT3_Sk-    QAQKHS---- ---------- ---------- ---KKAGATA KGKVGGRWK- ---------- ---------- ---------- ---------- ----------
SaTnT-a_Sk    ELSKFS---- ---------- ---------- ---KKGAAAR RRK------- ---------- ---------- ---------- ---------- ----------
SaTnT-e_Sk    ELSKFS---- ---------- ---------- ---KKGAAAR RRK------- ---------- ---------- ---------- ---------- ----------
SaTnT-l_Sk    ELSKFS---- ---------- ---------- ---KKGAAAR RRK------- ---------- ---------- ---------- ---------- ----------
SaTnT1_Sk-    HAQKFK---- ---------- ---------- ---KVHG--- KGKVGGRWK- ---------- ---------- ---------- ---------- ----------
SaTnT2_Sk-    DHQSAKGR-- ---------- -----G---- ---KGKMAGR LR-------- ---------- ---------- ---------- ---------- ----------
SaTnTi_Sk_    DHQ--RGS-- ---------- -----K---- ---ASKTSRG AKGKAGSLK- ---------- ---------- ---------- ---------- ----------
SpTnT1_sea    IQEEKLK--- ---------- ---------- --ASKSADGK IQKINIPFGE VNAEAD---- ---------- ---------- ---------- ----------
SpTnT2_sea    LQAAKVK--- ---------- ---------- --AAHSADGL IKKIALPFGE VAE------- ---------- ---------- ---------- ----------
SsTnT_Sk-f    ELSKFS---- ---------- ---------- ---KKGKTVR RK-------- ---------- ---------- ---------- ---------- ----------
StTnT1S_Sk    DHQSAKGR-- ---------- -----G---- ---KAKAVRR ---------- ---------- ---------- ---------- ---------- ----------
XlTnT2a_Ca    DHQKLKSK-- ---------- -----S---- ---SKGPRAG KGILGGRWK- ---------- ---------- ---------- ---------- ----------
XlTnT2b_Ca    DHQKLKSK-- ---------- -----S---- ---SKGPRAG KGILGGRWK- ---------- ---------- ---------- ---------- ----------
XlTnT3_Sk-    ELSKFS---- ---------- ---------- ---KKAAG-G KGKVGGRWK- ---------- ---------- ---------- ---------- ----------
```

# Figure S7 (p 6/6)

```
              ....|....| ....|....| ....|....| ....|....| ....|....| ....|....| ....|....| ....|....| ....|....| ....|....| ..
                     510        520        530        540        550        560        570        580        590
AmTnT_hone    ERPGKK---- -PGDPESPEG EEDVKAAAE- DEELEEPQFE EEEEEEEEEE EEEEEEGEKK EGEGEGEEEE EEEEEEEEEE EEEEEEEEEE EE
BfTnT1a_Bu    ---------- ---------- ---------- ---------- ---------- ---------- ---------- ---------- ---------- ---------- --
BfTnT1b_Bu    ---------- ---------- ---------- ---------- ---------- ---------- ---------- ---------- ---------- ---------- --
BflTnT_amp    ---------- ---------- ---------- ---------- ---------- ---------- ---------- ---------- ---------- ---------- --
BmTnT_Lepi    ERPGKK---- -KGELESPEG EEDVKAEP-- -EEDEEPQYE ------PEPE EEEEEV---- ------VEEV EEEEEEEEEE EEEEEEEEEE E-
BsTnT-c_Bo    ---------- ---------- ---------- ---------- ---------- ---------- ---------- ---------- ---------- ---------- --
BtTnT1_Sk-    ---------- ---------- ---------- ---------- ---------- ---------- ---------- ---------- ---------- ---------- --
BtTnT2_Car    ---------- ---------- ---------- ---------- ---------- ---------- ---------- ---------- ---------- ---------- --
BtTnT3_Sk-    ---------- ---------- ---------- ---------- ---------- ---------- ---------- ---------- ---------- ---------- --
CiTnT_Cion    ---------- ---------- ---------- ---------- ---------- ---------- ---------- ---------- ---------- ---------- --
CjTnT3_Sk-    ---------- ---------- ---------- ---------- ---------- ---------- ---------- ---------- ---------- ---------- --
CnTnT_Biva    EAAPAP---- ---------- ---------- ---------- ---------- ---------- ---------- ---------- ---EAEVAAE EE-------- --
Csp1TnT_po    AAAAAN---- ---------- ---------- ---------- ---------- ---------- ---------- ---------- ---AADEAEE REEEAEQVEE --
DmTnT_frui    ERPGKK---- -AGEPETPEG EEDAKADEDI VEDDEEVEEE VVEEEDEEAE EDEEEE---- ------EEEE EEEEEEEEEE EEEEEEEEEE EE
DpTnT_Daph    ERPGKK---- -KGDTDSPDG EE---AEPK- -DVDVDDFLE ------PPPP VVEEEE---- -------EQE EEEEEEEEEE EEEEEEEEEE E-
DrTnT1_Sk-    ---------- ---------- ---------- ---------- ---------- ---------- ---------- ---------- ---------- ---------- --
DrTnT2_Car    ---------- ---------- ---------- ---------- ---------- ---------- ---------- ---------- ---------- ---------- --
DrTnT3a_Sk    ---------- ---------- ---------- ---------- ---------- ---------- ---------- ---------- ---------- ---------- --
DrTnT3b_Sk    ---------- ---------- ---------- ---------- ---------- ---------- ---------- ---------- ---------- ---------- --
FcTnT2_Car    ---------- ---------- ---------- ---------- ---------- ---------- ---------- ---------- ---------- ---------- --
GgTnT1_Sk-    ---------- ---------- ---------- ---------- ---------- ---------- ---------- ---------- ---------- ---------- --
GgTnT2_Car    ---------- ---------- ---------- ---------- ---------- ---------- ---------- ---------- ---------- ---------- --
GgTnT3_Sk-    ---------- ---------- ---------- ---------- ---------- ---------- ---------- ---------- ---------- ---------- --
GmTnT_Sk-f    ---------- ---------- ---------- ---------- ---------- ---------- ---------- ---------- ---------- ---------- --
HcTnT_Hyla    ---------- ---------- ---------- ---------- ---------- ---------- ---------- ---------- ---------- ---------- --
HqTnT_Arac    ENPKNKQVSH IRRVDEDDED EIEPVFEPPK PEPPKPAPPA PKPAPVQQKQ EEEEEE---- ------EEEE EEEEEDEEEE EEEEEEEEEE EE
HrTnT-a_Ha    ---------- ---------- ---------- ---------- ---------- ---------- ---------- ---------- ---------- ---------- --
HrTnT-l_Ha    ---------- ---------- ---------- ---------- ---------- ---------- ---------- ---------- ---------- ---------- --
HroTnT_Hir    AE-------- ---------- ---------- ---------- ---------- ---------- ---------- ---------- ---------- ---------- --
HsTnT1_Sk-    ---------- ---------- ---------- ---------- ---------- ---------- ---------- ---------- ---------- ---------- --
HsTnT2_Car    ---------- ---------- ---------- ---------- ---------- ---------- ---------- ---------- ---------- ---------- --
HsTnT3_Sk-    ---------- ---------- ---------- ---------- ---------- ---------- ---------- ---------- ---------- ---------- --
MmTnT1_Sk-    ---------- ---------- ---------- ---------- ---------- ---------- ---------- ---------- ---------- ---------- --
MmTnT2_Car    ---------- ---------- ---------- ---------- ---------- ---------- ---------- ---------- ---------- ---------- --
MmTnT3_Sk-    ---------- ---------- ---------- ---------- ---------- ---------- ---------- ---------- ---------- ---------- --
MtTnT3_Sk-    ---------- ---------- ---------- ---------- ---------- ---------- ---------- ---------- ---------- ---------- --
OcTnT2_Car    ---------- ---------- ---------- ---------- ---------- ---------- ---------- ---------- ---------- ---------- --
OcTnT3_Sk-    ---------- ---------- ---------- ---------- ---------- ---------- ---------- ---------- ---------- ---------- --
RnTnT1_Sk-    ---------- ---------- ---------- ---------- ---------- ---------- ---------- ---------- ---------- ---------- --
RnTnT2_Car    ---------- ---------- ---------- ---------- ---------- ---------- ---------- ---------- ---------- ---------- --
RnTnT3_Sk-    ---------- ---------- ---------- ---------- ---------- ---------- ---------- ---------- ---------- ---------- --
SaTnT-a_Sk    ---------- ---------- ---------- ---------- ---------- ---------- ---------- ---------- ---------- ---------- --
SaTnT-e_Sk    ---------- ---------- ---------- ---------- ---------- ---------- ---------- ---------- ---------- ---------- --
SaTnT-l_Sk    ---------- ---------- ---------- ---------- ---------- ---------- ---------- ---------- ---------- ---------- --
SaTnT1_Sk-    ---------- ---------- ---------- ---------- ---------- ---------- ---------- ---------- ---------- ---------- --
SaTnT2_Sk-    ---------- ---------- ---------- ---------- ---------- ---------- ---------- ---------- ---------- ---------- --
SaTnTi_Sk_    ---------- ---------- ---------- ---------- ---------- ---------- ---------- ---------- ---------- ---------- --
SpTnT1_sea    ---------- ---------- ---------- ---------- ---------- ---------- ---------- ---------- ---------- ---------- --
SpTnT2_sea    ---------- ---------- ---------- ---------- ---------- ---------- ---------- ---------- ---------- ---------- --
SsTnT_Sk-f    ---------- ---------- ---------- ---------- ---------- ---------- ---------- ---------- ---------- ---------- --
StTnT1S_Sk    ---------- ---------- ---------- ---------- ---------- ---------- ---------- ---------- ---------- ---------- --
XlTnT2a_Ca    ---------- ---------- ---------- ---------- ---------- ---------- ---------- ---------- ---------- ---------- --
XlTnT2b_Ca    ---------- ---------- ---------- ---------- ---------- ---------- ---------- ---------- ---------- ---------- --
XlTnT3_Sk-    ---------- ---------- ---------- ---------- ---------- ---------- ---------- ---------- ---------- ---------- --
```

**Figure S7.** Alignment of troponin T sequences. Areas used for molecular phylogenetic analysis are boxed.
