# Supplementary material for: Muscle differentiation in a colonial ascidian: organisation, gene expression and evolutionary considerations
Source: BMC Dev Biol. 2009 Sep 8;9:48. doi: 10.1186/1471-213X-9-48 (PMC2753633; doi:10.1186/1471-213X-9-48)
Supplement: Additional file 8 — Figure S8. Pairwise scores calculated between TnTs of some deuterostomes compared to a rabbit fast skeletal muscle form. [file 1471-213X-9-48-S8.pdf]

	Tm site 1	Tm site 2	TnC	TnI	Total
**Ascidian BsTnT-c**	51	46	36	40	**47**
**Ascidian HrTnT-a**	58	45	36	40	**45**
**Amphioxus BflTnT**	46	27	28	60	**36**
**Sea urchin SpTnT2**	22	27	33	80	**23**

Figure S8. Pairwise scores calculated between TnTs of some deuterostomes compared to a rabbit fast skeletal muscle form.

The analysis is performed among $OcTnT_{2f}$ (rabbit fast skeletal TnT; GenBank:P02641-4) and the TnTs of *B. schlosseri* (BsTnT-c), *H. roretzi* (HrTnT-a), *Branchiostoma floridae* (BflTnT) and *Strongylocentrotus purpuratus* (SpTnT2). The considered region includes the probable interaction sites with the proteins of the contractile apparatus: tropomyosin interaction site 1 ($BsTnT\text{-}c^{63\text{-}143}$), site 2 ($BsTnT\text{-}c^{151\text{-}259}$), troponin C ($BsTnT\text{-}c^{221\text{-}260}$) and troponin I ($BsTnT\text{-}c^{216\text{-}220}$). For the other accession numbers see Figure 1.
